# A general model for metabolic scaling in self-similar asymmetric networks

**DOI:** 10.1371/journal.pcbi.1005394

**Published:** 2017-03-20

**Authors:** Alexander Byers Brummer, Van M. Savage, Brian J. Enquist

**Affiliations:** 1 Department of Physics, University of Arizona, Tucson, Arizona, United States of America; 2 Department of Ecology and Evolutionary Biology, University of Arizona, Tucson, Arizona, United States of America; 3 Department of Biomathematics, UCLA David Geffen School of Medicine, Los Angeles, California, United States of America; 4 Department of Ecology and Evolutionary Biology, UCLA, Los Angeles, California, United States of America; 5 Santa Fe Institute, Santa Fe, New Mexico, United States of America; EMBL-Heidelberg, GERMANY

## Abstract

How a particular attribute of an organism changes or scales with its body size is known as an allometry. Biological allometries, such as metabolic scaling, have been hypothesized to result from selection to maximize how vascular networks fill space yet minimize internal transport distances and resistances. The West, Brown, Enquist (WBE) model argues that these two principles (space-filling and energy minimization) are (i) general principles underlying the evolution of the diversity of biological networks across plants and animals and (ii) can be used to predict how the resulting geometry of biological networks then governs their allometric scaling. Perhaps the most central biological allometry is how metabolic rate scales with body size. A core assumption of the WBE model is that networks are symmetric with respect to their geometric properties. That is, any two given branches within the same generation in the network are assumed to have identical lengths and radii. However, biological networks are rarely if ever symmetric. An open question is: Does incorporating asymmetric branching change or influence the predictions of the WBE model? We derive a general network model that relaxes the symmetric assumption and define two classes of asymmetrically bifurcating networks. We show that asymmetric branching can be incorporated into the WBE model. This asymmetric version of the WBE model results in several theoretical predictions for the structure, physiology, and metabolism of organisms, specifically in the case for the cardiovascular system. We show how network asymmetry can now be incorporated in the many allometric scaling relationships via total network volume. Most importantly, we show that the 3/4 metabolic scaling exponent from Kleiber’s Law can still be attained within many asymmetric networks.

## Author summary

We present a model for incorporating geometrically asymmetric branching into biological resource distribution networks. Our work shows how space-filling and fluid flow principles constrain allowed branching morphologies within the context of our model. Simultaneously, we demonstrate that there is a wide range of asymmetrically branching network architectures that still give rise to 3/4 metabolic scaling exponents.

## Introduction

One of the pervasive characteristics of biology is that the metabolic rate, *B*, of an organism scales with its body mass, *M*. When viewed across several orders of magnitude this relationship is approximated by the power law,

$$B=B_{0}M^{\theta }$$ (1)

where *B_0_* is a normalization constant, and *θ*, the allometric scaling exponent, tends to cluster around the value of 3/4 [1–5]. This metabolic scaling relationship spans more than twenty five orders of magnitude in mass, from respiratory complexes at 10^-18^ grams to the largest mammals at 10^7^ grams [6]. The mechanistic origin of the 3/4 scaling exponent has been one of the longest running debates in biology [1, 7–11].

**Fig 1:**
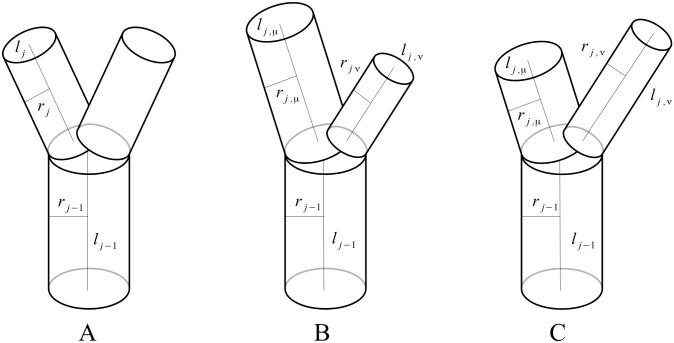
Diagram of symmetric and asymmetric bifurcations. *Symmetric branching* (**A**) is characterized by every branch within a given generation j having equal values of radius and length. *Positive asymmetry* (**B**) is such that one child branch is larger than the other in both radius and length. *Negative asymmetry* (**C**) is such that one child branch has a larger radius and shorter length while the other has a smaller radius and greater length.

The West, Brown, and Enquist (WBE) model offers an alternative hypothesis for the origin of allometric scaling exponents in biology, in particular the 3/4 exponent of metabolic scaling [9]. The WBE model shows theoretically how numerous allometric scaling exponents are the result of a repeated, or self-similar, branching architecture. The pattern to be repeated consists of one parent branch and two or more child branches and is shown in Fig 1**A**. This pattern is sometimes referred to as a branching node, or generator, and depending on the geometric properties constitutes space-filling fractal [9, 12]. One of the appeals of the WBE model is that, with relatively few starting assumptions, it predicts numerous biological allometric scaling relationships within and across diverse organisms (such as plants and animals).

The WBE model for biological resource distribution networks has seen extensive application across a wide range of scientific disciplines. Within the original context of cardiovascular networks it has been used to further understanding regarding tumor growth [13], the temperature dependence of basic metabolic function on allometric scaling [14], and the limits of its applicability under the constraint of finite-size networks [15]. Beyond cardiovascular networks, the WBE model has aided in the advancement of understanding respiratory networks, plant vascular networks, ecosystem dynamics, and has even served as motivation for the study of other observed allometries, such as the form and function of major metropolitan cities [9, 12, 16–20]. Of note in all of these extensions is the underlying principle that the network is geometrically symmetric.

While the WBE model predicts the metabolic scaling exponent of *θ* = 3/4, several have questioned its assumptions and ability to explain the observed variation in metabolic scaling exponents as well as capture the biological variation in organism form and function [10, 21, 22]. In response, several studies have pointed to extensions of the WBE model to explain variation in biological allometries and have shown how relaxation of some of its assumptions can indeed account for observed variation in biological scaling [14, 15, 23, 24]. Nonetheless, it still is unclear if the assumptions of how the WBE model characterizes hierarchical branching networks in biology hold across the diversity of life. A fundamental assumption of the theory is that vascular networks exhibit symmetric branching, where every branch in a given generation has identical geometric properties (namely radius and length). Branching symmetry is rarely, if ever, the case within biology where vascular networks are observed to branch asymmetrically [25–31].

Existing in nature are several biological examples of observed asymmetric branching patterns that help to motivate our approach. In an early study focusing on casts of the human heart, Zamir introduced a characterization of two branching strategies based on differences in the scaling of vessel diameters. By measuring the differences in sibling branch radius (Fig 1) with respect to the frequency of branching events, one can distinguish between so-called *distributing* vessels and *delivering* vessels. The former, which act to transport blood toward myocardial zones, correspond to large differences in sibling radii; whereas the latter type, responsible for transporting blood into the myocardial zones, correspond to small differences in radius [26]. Recently, studies of the interplay between genetics and vascular morphology have led to additional branching strategy classifications. In particular, a clear hierarchical pattern is formed during the development of the mouse lung that starts with *domain*-type branching (“scaffolding”) and transitions to either *planar*-type branching (“edge” features), or *orthogonal*-type branching (“surfaces” and “interiors”). The qualitatively defining characteristics of these branching types are as follows: in *domain*-type branching there are large differences between sibling branch lengths (Fig 1); in *planar* and *orthogonal* there are little to no differences between sibling branch characteristics, but in the former all branches exist in the same geometric plane, whereas in the latter a rotation of roughly *π*/2 occurs at every branching event [28]. At the whole-organism scale, the Zebrafish has become a remarkable standard for the study of vascular form and development, owing to the translucent nature of its skin. A particularly stark feature of the Zebrafish vasculature is the presence of patterned asymmetric branching in the intersegmental vessels originating from the dorsal vein [29].

The rarity of symmetric branching networks in biology violates a core assumption of the WBE model and questions its validity. As all of the predictions of the WBE model are derived from a symmetric network, an important question is, does incorporating asymmetry impact the predictions of the model? However, the fact that approximate 3/4 scaling is observed within and across organisms characterized by asymmetric branching suggests that perhaps the 3/4 scaling may not depend on the degree of asymmetry exhibited [32].

The study of asymmetric branching patterns itself has a long history. At its earliest, asymmetric branching was included in some of the first uses of energy minimization procedures in studying the cardiovascular system [25, 33–35]. In these early studies, the energy minimization procedures were imposed at the nodal level instead of the whole network level, where a node is defined by the occurrence of a branching. Thus, the influence of branching asymmetry on biological scaling was not explored. More recently, asymmetric branching has been studied within the context of leaf venation patterns, river branching, and both cardio- and plant vascular metabolic rates [32, 36]. Nonetheless, these studies do not provide detailed analytic treatments of the role of asymmetry in governing the scaling behavior of biological networks.

Here, we present a more general model for the origin of allometric scaling laws. We present a theoretical foundation for incorporating asymmetric branching in the WBE framework for the cardiovascular system. As a result, this model can better capture the diversity of network morphologies observed in biology by allowing for generational variation in the values of branch radii and lengths between sibling branches. Our model shows how different scaling exponents, such as 2/3 and 1, can be associated with different levels of asymmetry, where 2/3 is the value associated with the idea that metabolic rate is limited by the ability of an organism to dissipate heat [37], and 1 is the value associated with isometric scaling. We also make predictions regarding a generational transition in asymmetry type that is associated with the transition in fluid flow from pulsatile to constant. Lastly, we show that there exists a wide range of asymmetric network morphologies that result in 3/4 metabolic scaling.

## Models

### Defining asymmetry

Our work starts with the same assumptions outlined in Ref. [9, 15]. However, we relax the assumption that child branches arising from a parent branch have equivalent lengths and radii (Fig 1**A**). As shown in Fig 1 there are two possible asymmetric network types. A *positive* asymmetry network is one in which the larger radius is paired with the larger length and the smaller radius with the smaller length (Fig 1**B**). A *negative* asymmetry network is one in which the larger radius is paired with the smaller length and the smaller radius is paired with the larger length (Fig 1**C**).

For any pair of two sibling branches, the asymmetric radii (*r_j,*μ*_, r_j,*ν*_*) and lengths (*l_j,*μ*_, l_j,*ν*_*) can be expressed in terms of their averages (*r_j_, l_j_*) and differences (Δ*r_j_*, Δ*l_j_*) as,

$$\begin{array}{cc} l_{j,\mu }=l_{j}+\Delta l_{j} & \;\;l_{j,\nu }=l_{j}-\Delta l_{j}\\ r_{j,\mu }=r_{j}+\Delta r_{j} & \;\;r_{j,\nu }=r_{j}-\Delta r_{j} \end{array}$$ (2)

where,

$$\begin{array}{cc} l_{j}=\frac{l_{j,\mu }+l_{j,\nu }}{2} & \;\;\Delta l_{j}=\frac{l_{j,\mu }-l_{j,\nu }}{2}\\ r_{j}=\frac{r_{j,\mu }+r_{j,\nu }}{2} & \;\;\Delta r_{j}=\frac{r_{j,\mu }-r_{j,\nu }}{2} \end{array}$$ (3)

Here the subscript *j* denotes the branching generation within the network, and the subscripts *μ* and *ν* distinguish the two child branches, as shown in Fig 1. In this formalism, the differences Δ*l*_*j*_ and Δ*r*_*j*_ can vary from positive to negative, and in so doing allow for smooth transitions between the two different asymmetry types. For example, in Fig 1**B**, Δ*r*_*j*_ > 0 and Δ*l*_*j*_ > 0, which corresponds to *positive asymmetric* branching, whereas in Fig 1**C**, Δ*r*_*j*_ > 0 but Δ*l*_*j*_ < 0, which corresponds to *negative asymmetric* branching. Note that, due to the definitions in Eq (2), the described examples are effectively identical to the scenarios in which Δ*r*_*j*_ < 0 and Δ*l*_*j*_ < 0 for *positive asymmetry*, and Δ*r*_*j*_ < 0 but Δ*l*_*j*_ > 0 for *negative asymmetry*.

Next, we define the scale factors between the physical child and parent dimensions,

$$\begin{array}{cc} \gamma_{j,\mu }=\frac{l_{j+1,\mu }}{l_{j}} & \;\;\gamma_{j,\nu }=\frac{l_{j+1,\nu }}{l_{j}}\\ \beta_{j,\mu }=\frac{r_{j+1,\mu }}{r_{j}} & \;\;\beta_{j,\nu }=\frac{r_{j+1,\nu }}{r_{j}} \end{array}$$ (4)

Using our definitions for the big and small radii and lengths from Eq (2), and introducing the *average* scale factors *β*_*j*_ = *r*_*j*+1_/*r*_*j*_ and *γ*_*j*_ = *l*_*j*+1_/*l*_*j*_, and the *difference* scale factors Δ*β*_*j*_ = Δ*r*_*j*+1_/*r*_*j*_ and Δ*γ*_*j*_ = Δ*l*_*j*+1_/*l*_*j*_, we can express the quantities in Eq (4) as,

$$\begin{array}{cc} \gamma_{j,\mu }=\gamma_{j}+\Delta \gamma_{j} & \;\;\gamma_{j,\nu }=\gamma_{j}-\Delta \gamma_{j}\\ \beta_{j,\mu }=\beta_{j}+\Delta \beta_{j} & \;\;\beta_{j,\nu }=\beta_{j}-\Delta \beta_{j} \end{array}$$ (5)

**Fig 2:**
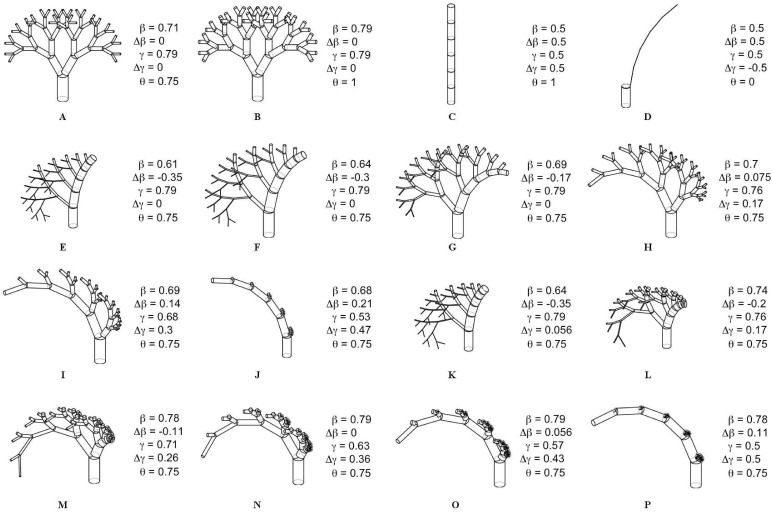
Rendering of Selected Networks. An assortment of networks are presented with associated *average* (*β*, *γ*) and *difference* (Δ*β*, Δ*γ*) scale factors, and metabolic scaling exponents (*θ*). Note that in all of these cases there is no switching of asymmetry type either within or across generations, and the scale factors are assumed to be constant both within and across branching generations. Networks (**A**) and (**B**) represent the symmetric limits under the constraints associated with pulsatile flow (Eqs (16) and (17)) and constant flow (Eqs (20) and (21)), respectively. Networks (**C**) and (**D**) represent the two extreme asymmetric limits. Networks (**E**) through (**J**) exhibit varying degrees of branching asymmetry while satisfying the constraints associated with pulsatile flow. Each of these tree networks are represented as points that fall along the 3/4 metabolic scaling contour as shown in Fig 4. Networks (**I**) through (**P**) satisfy constant laminar flow, and they also fall along the 3/4 metabolic scaling contour as shown in Fig 5.

The *difference* scale factors will come to represent the measures of asymmetry exhibited within a network. Specifically, Δ*β*_*j*_ = Δ*γ*_*j*_ = 0 represents maximally symmetric branching (see Fig 2**A** and 2**B**), and Δ*β*_*j*_ ≈ ±*β*_*j*_ and Δ*γ*_*j*_ ≈ ±*γ*_*j*_ represent maximally asymmetric branching (see Fig 2**C** and 2**D**).

The benefits of defining asymmetry in the manner described above are twofold. At the theoretical level this will allow us to express all results in terms of contributions due to symmetrically (*β* and *γ*) and asymmetrically (Δ*β* and Δ*γ*) defined quantities. Furthermore, we are not restricted to any particular pair of values for the scale factors (e.g. *γ*_*μ*_ and *γ*_*ν*_). That is, any pair of scale factors can be described by their *average* and *difference* scale factors. At the experimental level this allows for an easy way to measure asymmetry. Traditionally, in studies conducted under the symmetric paradigm, the values of the scale factors are calculated as nodal averages, or alternatively, distributions of the scale factors for entire networks are collected, where the means of the distributions are meant to represent the average value of the scale factor for that network [30, 36, 38, 39].

An alternative formulation for characterizing asymmetry can also be made. In this approach, the physical scale factors are expressed in terms of the *symmetric* WBE scale factors and perturbations from those values. Starting with an analogous form of Eq (2), we can write the physical lengths and radii of a pair of sibling branches as,

$$\begin{array}{cc} l_{j,\mu }={\tilde{l}}_{j}+\Delta {\tilde{l}}_{j,\mu } & \;\;l_{j,\nu }={\tilde{l}}_{j}-\Delta {\tilde{l}}_{j,\nu }\\ r_{j,\mu }={\tilde{r}}_{j}+\Delta {\tilde{r}}_{j,\mu } & \;\;r_{j,\nu }={\tilde{r}}_{j}-\Delta {\tilde{r}}_{j,\nu } \end{array}$$ (6)

We can express the above in terms of scale factors using Eq (4), to arrive at,

$$\begin{array}{cc} \gamma_{j,\mu }={\tilde{\gamma }}_{\text{WBE}}+\Delta {\tilde{\gamma }}_{j,\mu } & \;\;\gamma_{j,\nu }={\tilde{\gamma }}_{\text{WBE}}-\Delta {\tilde{\gamma }}_{j,\nu }\\ \beta_{j,\mu }={\tilde{\beta }}_{\text{WBE}}+\Delta {\tilde{\beta }}_{j,\mu } & \;\;\beta_{j,\nu }={\tilde{\beta }}_{\text{WBE}}-\Delta {\tilde{\beta }}_{j,\nu } \end{array}$$ (7)

where ${\tilde{\beta }}_{\text{WBE}}$ and ${\tilde{\gamma }}_{\text{WBE}}$ are the *symmetric* WBE scale factors and $\Delta {\tilde{\gamma }}_{j,\mu }$, $\Delta {\tilde{\gamma }}_{j,\nu }$, $\Delta {\tilde{\beta }}_{j,\mu }$ and $\Delta {\tilde{\beta }}_{j,\nu }$ are subsequently called the *symmetric-difference* scale factors. Furthermore, ${\tilde{\beta }}_{\text{WBE}}={\left(1/2\right)}^{1/2}$ for pulsatile flow and (1/2)^1/3^ for constant laminar flow, and ${\tilde{\gamma }}_{\text{WBE}}={\left(1/2\right)}^{1/3}$ for both types of flow, while the *symmetric-difference* scale factors are free to vary. While this approach is beneficial in that it allows for results to be expressed strictly in terms of deviations from the *symmetric* WBE results, it does not as easily distinguish between positive and negative type asymmetry, and in certain circumstances may even obscure the presence (or absence) of asymmetry all together. For example, should both child branches have physical length scale factors of 0.8, then the *symmetric-difference* length scale factors would have non-zero values of $\Delta {\tilde{\gamma }}_{j,\mu }=0.8-{\tilde{\gamma }}_{\text{WBE}}$ and $\Delta {\tilde{\gamma }}_{j,\nu }={\tilde{\gamma }}_{\text{WBE}}-0.8$. For a further exploration of this approach, see S3 Text.

## Results

**Fig 3:**
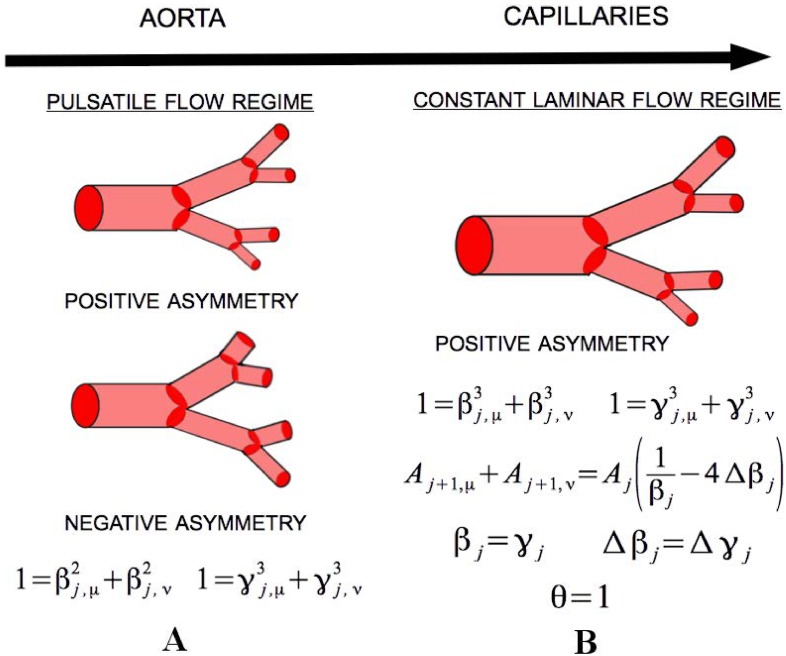
Results of Minimizing Network Energy Loss. In the pulsatile flow regime (**A**) either network asymmetry type is allowed, but both must follow the area-preserving and space-filling principles at the nodal level, as per Eqs (16) and (17). In this regime switching between asymmetry types can occur both within and across generations. In the constant laminar flow regime (**B**) only positive network asymmetry is predicted, and the scale factors must follow the space-filling and Murray’s Law relations, as per Eqs (20) and (21). We also find that the asymmetric version of Murray’s Law allows for an expression of the total cross-sectional area, *A*, of any two child branches in terms of the parent area and the *average* and *difference* scale factors, showing that asymmetric branching allows for a toggling between branching with increasing area and constant area. Lastly, the *average* radius and length scale factors are predicted to be equal, as well as the *difference* radius and length scale factors. When combining these two results with the space-filling and Murray’s Law expressions, we can make a strict prediction of the metabolic scaling exponent *θ* = 1 for the constant laminar flow regime.

Of significant interest is examining the effect of asymmetric branching on the predicted values of the metabolic scaling exponent *θ*. As we are still working under the main principles of the *symmetric* WBE model, we focus our study on the class of asymmetric networks that minimize energy-loss during fluid transport, and that have a branching architecture that can be characterized as a space-filling fractal. These principles act to constrain the allowed values of the scale factors by forcing them to covary according to specific mathematical relationships at the nodal, or generational, level. We do this for networks consisting of either pulsatile flow, or constant laminar flow. The specifics of the techniques for minimizing energy loss and imposing space-filling are described in our **Methods** section. The results are summarized in Fig 3, and presented at length in the **Nodal level results** section.

### Metabolic scaling

#### Deriving the exponent

Here we outline the calculation for the metabolic scaling exponent for asymmetric networks under the following assumptions:

1. The networks are space-filling fractals that minimize energy-loss such that Eqs (16) and (17) are satisfied for pulsatile flow, and Eqs (20) and (21) are satisfied for constant laminar flow.
2. No mixing of asymmetry type occurs either across, or within, all branching generations.
3. The scale factors are assumed to be constant both across, and within, all branching generations.
4. Truncation effects associated with the terminal capillary size are neglected. Specifically, we assume *N*_*C*_ ≈ 2^*N*^, where *N*_C_ is the number of terminal capillaries, and *N* the total number of generations.

While biologically realistic organisms may exhibit some variation in asymmetry type within and across generations, non-constant scale-factors, and truncation due to finite-size effects, our assumptions still provide general insight into how asymmetric branching influences the metabolic scaling exponent as a first order approximation.

Similar to the *symmetric* WBE model, the metabolic scaling exponent *θ* can be related to the whole-organism mass *M* by,

$$\theta =\frac{\ln(N_{C})}{\ln(M/M_{0})}$$ (8)

where *M*_0_ is a normalization constant, and *N*_C_ is the total number of capillaries [9, 15]. This relationship is a result of the core WBE assumption that many organism properties scale allometrically with organism vasculature. In the context of the cardiovascular system, we are interested in the scaling of the number of terminal branches or capillaries, *N*_C_, with body mass, *M*, or *N*_*C*_ ∝ *M*^*θ*^. Furthermore, it can be shown that the whole-organism mass is proportional to the total network volume, *M* ∝ *V*_*TOT*_, which comes as a result of the energy-loss minimization procedure (see S1 Text).

To derive an expression for the metabolic scaling exponent for an asymmetric network, we must begin with the total volume of the network. In our **Methods** section we derive such a relationship and present here the result. Having utilized assumptions 1–4, the total volume of a vascular network can be approximated as,

$$V_{TOT}\approx \frac{N_{C}V_{C}}{{\left[\beta_{\mu }^{2}\gamma_{\mu }+\beta_{\nu }^{2}\gamma_{\nu }\right]}^{N}}$$ (9)

where $V_{C}=\pi r_{C}^{2}l_{C}$ is the volume of a capillary. Using *M* ∝ *V*_*TOT*_ to substitute the above expression into Eq (8) gives,

$$\theta \approx \frac{\ln(2)}{\ln\left(2\right)-\ln\left(\beta_{\mu }^{2}\gamma_{\mu }+\beta_{\nu }^{2}\gamma_{\nu }\right)}$$ (10)

Up until this point we have not chosen a particular characterization of asymmetry with which to work. That is, either of the *average/difference* or the *symmetric/difference* formalisms may be substituted for the physical scale-factors. We will adopt the *average/difference* formalism. From the definitions for the scale factors in Eq (4), we can express the above in terms of the *average* and *difference* scale factors,

$$\theta \approx -{\left\{\frac{\ln(\beta^{2}\gamma )}{\ln(2)}+\frac{\ln\;\left(1+\frac{2\Delta \beta \Delta \gamma }{\beta \gamma }+\frac{\Delta \beta^{2}}{\beta^{2}}\right)}{\ln(2)}\right\}}^{-1}$$ (11)

where the first term in the above expression yields the 3/4 metabolic scaling exponent result in the symmetric limit that *β* = (1/2)^1/2^, *γ* = (1/2)^1/3^, and Δ*β* = Δ*γ* = 0.

**Fig 4:**
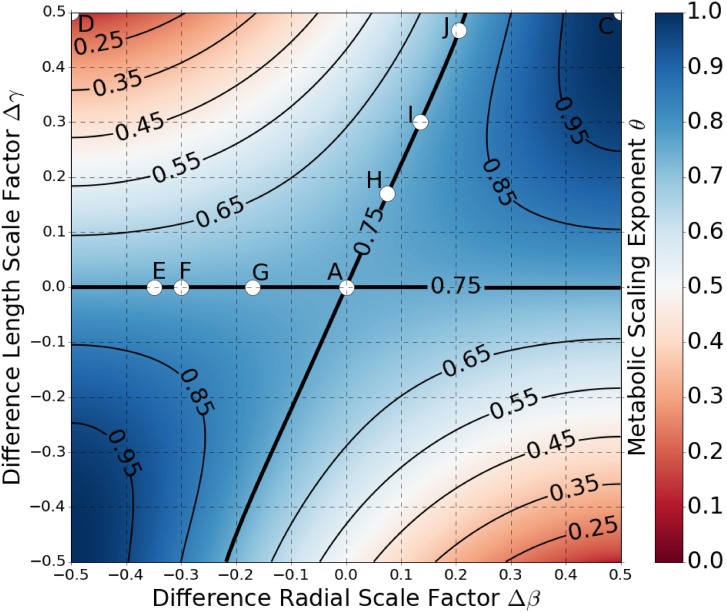
Colormap of Metabolic Scaling Exponent for Pulsatile Flow. The metabolic scaling exponent is graphed as a function of the *difference* scale factors Δ*γ* and Δ*β*, and is shown to range in value from 0 to 1. The scale factors are such that the networks are space-filling fractals that minimize energy-loss from resource transport, as dictated by Eqs (16) and (17). Positive asymmetry is graphed in the first and third quadrants, and negative asymmetry in the second and fourth quadrants. Contours of constant values of the metabolic scaling exponent are plotted in bold. The points labelled **A**, **C**, **D**, and **E**—**J** correspond to the rendered trees found in Fig 2.

**Fig 5:**
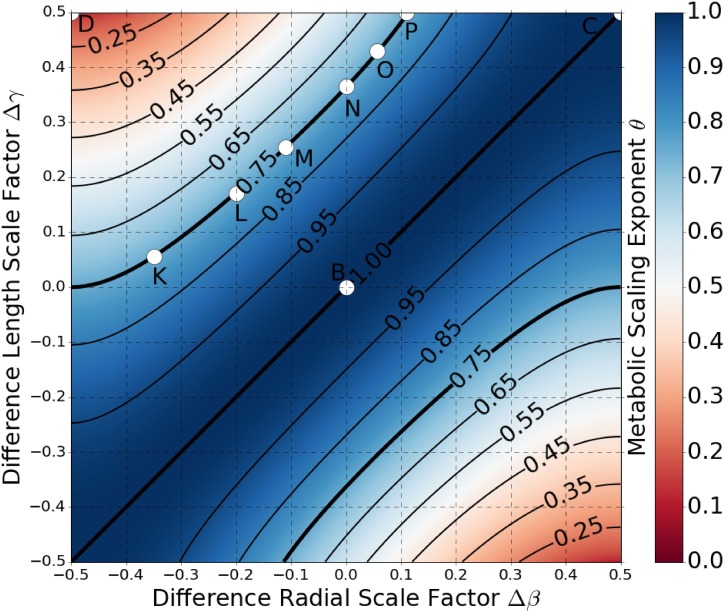
Colormap of Metabolic Scaling Exponent for Constant Laminar Flow. The metabolic scaling exponent is graphed as a function of the *difference* scale factors Δ*γ* and Δ*β*, and is shown to range in value from 0 to 1. The scale factors are such that the networks are space-filling fractals that minimize energy-loss from resource transport, as dictated by Eqs (20) and (21). Positive asymmetry is graphed in the first and third quadrants, and negative asymmetry in the second and fourth quadrants. Contours of constant values of the metabolic scaling exponent are plotted in bold. The points labelled **B**, **C**, **D**, and **K**—**P** correspond to the rendered trees found in Fig 2.

Eq (11) is convenient in that it clearly highlights the symmetric and asymmetric contributions to the metabolic scaling exponents. However, it is limited in that it does not make transparent the explicit covariation between the *average* and *difference* scale factors that results from our first assumption. To examine the metabolic scaling exponent in this scenario, we have graphed Eq (11) in Figs (4) and (5) for when the *average* and *difference* scale factors are constrained to satisfy Eqs (16) and (17) for pulsatile flow and Eqs (20) and (21) for constant laminar flow. In these graphs the value of the metabolic scaling exponent is plotted as a function of the *difference* scale factors Δ*β* and Δ*γ*. Positive asymmetry is presented in the first and third quadrants where Δ*β* and Δ*γ* are both positive (or both negative), and negative asymmetry is in the second and fourth quadrants where Δ*γ* is positive (or negative), but Δ*β* is negative (or positive).

In both Figs (4) and (5) we can see that the metabolic scaling exponent depends heavily on asymmetry, ranging in value from 0 to 1. These limits are of interest when considering metabolic rate per mass, where sub-linear scaling indicates that more massive organisms are more efficient energy converters per mass. Thus, a scaling exponent of 1 represents linear scaling and zero advantage in greater size, while an exponent of 0 represents the extreme limit of sub-linear scaling and maximal advantage in greater size.

It is important to connect these limits in organismal metabolic performance to physical network manifestation. The network corresponding to the limit of *θ* = 0 is shown in Fig 2**D**. The values of the physical scale factors here are: *β*_*μ*_ = 1, *β*_*ν*_ = 0, *γ*_*μ*_ = 0, and *γ*_*ν*_ = 1. These values produce a highly non-physical network due to the fact that at every node one of the child branches has zero length, while the other child branch has zero diameter. On the other hand, the network corresponding to the limit of *θ* = 1 is shown in Fig 2**C**. Here the values of the physical scale factors are: *β*_*μ*_ = 1, *β*_*ν*_ = 0, *γ*_*μ*_ = 1, and *γ*_*ν*_ = 0. This limit produces a network that takes on the shape of one singular cylinder with constant radius.

#### Metabolic scaling in a pulsing flow network

The extent to which asymmetry in radius or length can influence the metabolic scaling exponent for values of *θ* between 0 and 1 varies for each of the cases. For the region of the cardiovascular network characterized by pulsatile flow, we see that 3/4 metabolic scaling can be maintained under the presence of asymmetric branching as long as the network is exhibiting either positive asymmetry, or there is zero length asymmetry, as indicated by the 3/4 contour line. That is, negative branching asymmetry precludes values of 3/4 for the metabolic scaling exponent. For the case of asymmetry in both length and radius, the 3/4 contour line is constrained to a domain of *difference* scale factors of Δ*β* between 0.0 and approximately 0.21, and Δ*γ* between 0.0 and 0.5. This suggests that 3/4 metabolic scaling is robust to maximal variation in the physical length scale factors (*γ*_*μ*_ or *γ*_*ν*_ within [0, 1]), as long as the variations in physical diameter scale factors (*β*_*μ*_ or *β*_*ν*_) are restricted to the range of approximately [0.47, 0.89]. While asymmetry in scale factors has not been directly measured, these qualitative differences in variation in the scale factors have been observed in cardiovascular data both for the human head and torso as well as for mouse lungs [30, 31].

For a better understanding of what particular values of scale factors imply in terms of realized network morphology, we present in Fig 2 several networks that fall along the 3/4 contour. Additionally, in S1 Video is a continuous animation of the evolution of asymmetric networks along the 3/4 contour.

For negative asymmetry the metabolic scaling exponent can only decrease from 3/4 upon the introduction of asymmetric branching, with maximal asymmetry leading to a value of 0 for the metabolic scaling exponent. This is counter to the case of positive asymmetry, where the metabolic scaling exponent can either increase or decrease from 3/4 depending on the particular values of the scale factors.

The primary dependence of the metabolic scaling exponent on asymmetry in length can be better seen by using the alternative characterization of asymmetry presented in Eq (7). This dependence is obscured from view in the *average/difference* formalism due to the fact that we are not explicitly incorporating the covariation between the *average* and *difference* scale factors. However, using the alternative formalism for asymmetry we can substitute into Eq (10) $\beta_{\nu }^{2}=1-\beta_{\mu }^{2}$ and $\gamma_{\nu }={\left(1-\gamma_{\mu }^{3}\right)}^{1/3}$ to explicitly incorporate the constraints due to energy minimization and space-filling. Using the definitions $\beta_{\mu }={\tilde{\beta }}_{WBE}+\Delta {\tilde{\beta }}_{\mu }$ and $\gamma_{\mu }={\tilde{\gamma }}_{WBE}+\Delta {\tilde{\gamma }}_{\mu }$, and with some rearrangement, we can arrive at the following alternative expression for the metabolic scaling exponent,

$$\theta_{\mu \mu }=-\left\{\frac{\ln({\tilde{\beta }}_{\text{WBE}}^{2}{\tilde{\gamma }}_{\text{WBE}})}{\ln(2)}+\frac{1}{\ln(2)}\ln\;\left[\frac{1}{2{\tilde{\beta }}_{\text{WBE}}^{2}}\;{\left(\frac{1}{{\tilde{\gamma }}_{\text{WBE}}^{3}}-\;{\left(1+\frac{\Delta {\tilde{\gamma }}_{\mu }}{{\tilde{\gamma }}_{\text{WBE}}}\right)}^{3}\right)}^{1/3}\right.\right.{\left.\left.+\frac{1}{2}\;{\left(1+\frac{\Delta {\tilde{\beta }}_{\mu }}{{\tilde{\beta }}_{\text{WBE}}}\right)}^{2}\left(\left(1+\frac{\Delta {\tilde{\gamma }}_{\mu }}{{\tilde{\gamma }}_{\text{WBE}}}\right)-{\left(\frac{1}{{\tilde{\gamma }}_{\text{WBE}}^{3}}-\;{\left(1+\frac{\Delta {\tilde{\gamma }}_{\mu }}{{\tilde{\gamma }}_{\text{WBE}}}\right)}^{3}\right)}^{1/3}\right)\right]\right\}}^{-1}$$ (12)

In Eq (12) we can now see that variation in the metabolic scaling exponent depends primarily on variations in length. Indeed, we can now show that the metabolic scaling exponent is actually invariant with respect to asymmetric branching in vessel radii as long as there does not exist any asymmetric branching in vessel lengths. To see this, note that the argument of the logarithm within the square brackets can be shown to reduce to ln[1] = 0 when setting $\Delta {\tilde{\gamma }}_{\mu }=0$ (or Δ*γ* = 0) and having substituted the definitions for the *symmetric* WBE scale factors, ${\tilde{\beta }}_{\text{WBE}}=1/2^{1/2}$ and ${\tilde{\gamma }}_{\text{WBE}}=1/2^{1/3}$. Consequently, the only term left is $\ln\left({\tilde{\beta }}_{\text{WBE}}^{2}{\tilde{\gamma }}_{\text{WBE}}\right)/\ln\left(2\right)$, which has no dependence at all on the asymmetry of vessel radii, $\Delta {\tilde{\beta }}_{\mu }$ (or Δ*β*), which we have left unspecified. In fact, this remaining term is exactly equal to 3/4 upon substitution of ${\tilde{\beta }}_{\text{WBE}}=1/2^{1/2}$ and ${\tilde{\gamma }}_{\text{WBE}}=1/2^{1/3}$.

It should be pointed out that, given the four possible combinations of substituting for *β*_*μ*_, *β*_*ν*_, *γ*_*μ*_, and *γ*_*ν*_, there are in fact four possible ways of expressing the metabolic scaling exponent under this formalism. However, the differences between these four expressions is primarily in the sign of the *symmetric-difference* scale factors, and their associated limits, which do not influence the general dependence of the metabolic scaling exponent on asymmetry in length or radius. These differences do influence the specific graph of the metabolic scaling exponent, or in this case the four graphs associated with the four possible substitutions, which is why we chose to present the dependence of the metabolic scaling exponent on asymmetric branching using the *average/difference* formalism as we can visualize the full extent of asymmetric branching in one single graph. For further discussion on these substitutions see S3 Text.

#### Metabolic scaling in a constant laminar flow network

For the constant laminar flow network the metabolic scaling exponent values are symmetric about the line Δ*β* = Δ*γ*, while along that line it maintains the exact value of *θ* = 1. This symmetry about the line Δ*β* = Δ*γ* is due to the fact that both sets of radial and length scale factors, (*β*, Δ*β*) and (*γ*, Δ*γ*), are governed by the same mathematical expressions, Eqs (20) and (21). Interestingly enough, in the constant laminar flow regime a prediction of *θ* = 1 for the value of the metabolic scaling exponent can be made. This results from the minimization of energy loss due to friction and adherence to space-filling, represented by Eqs (18)–(21), and is proven here. Starting from Eq (11) and substituting *β* = *γ* and Δ*β* = Δ*γ* gives,

$$\theta =-{\left\{\frac{\ln(\gamma^{3})}{\ln(2)}+\frac{\ln(1+\frac{3\Delta \gamma^{2}}{\gamma^{2}})}{\ln(2)}\right\}}^{-1}$$ (13)

From Eq 20 we can derive the following relationship, 3Δ*γ*^2^/*γ*^2^ = 1/2*γ*^3^ − 1, and substitute it into the above expression to find a predicted value of *θ* = 1 for the constant laminar flow, positive asymmetry network. Furthermore, this is the same value as that predicted by the *symmetric* version of the WBE model [9].

#### Metabolic scaling in a network with a transition from pulsing to constant laminar flow

So far we have limited our analysis to networks exhibiting not just only one type of fluid flow, but also being governed by only one set of constant scale factors across each generation. A natural question to investigate is how our predictions change upon the introduction of a transition in fluid flow type from pulsing to constant laminar flow. In the *symmetric* WBE model, incorporating the fluid flow transition leads to predictions for the metabolic scaling exponent that fall between 3/4 and 1, depending on which generation the transition occurs [9, 15].

Under the *symmetric* WBE model, incorporating a transition in flow type starts by identifying at which generation the two different resistances to fluid flow become equal to one another from a parent branch to a child branch. This approach is greatly simplified by the *symmetric* branching assumption, as the equality of resistance types from parent-to-child is guaranteed to occur at the same generation throughout the network. Modeling a transition in an *asymmetric* network in such a manner is greatly complicated by the fact that such parent-to-child equalities of resistances will occur at different generations. We take an alternative approach to the problem that, while less physically motivated than matching fluid resistances, still provides for general insight regarding the presence of a transition in flow type.

In our approach, we forego the requirement that the transitioning generation is determined by the resistances, and instead simply impose a transition in fluid flow type at a given generation *M*. Thus, for all generations less than *M*, the network has pulsing type flow and is governed by a set of constant scale factors {*β*_<,*μ*_, *γ*_<,*μ*_, *β*_<,*ν*_, *γ*_<,*ν*_} (where the subscripted less than sign signifies that these are pre-transition scale factors). For all generations greater than *M*, the network has constant laminar type flow and is governed by a new set of constant scale factors {*β*_>,*μ*_, *γ*_>,*μ*_, *β*_>,*ν*_, *γ*_>,*ν*_}. We can approximate the total volume of such a network in a manner similar to Eq (9),

$$V_{TOT}\approx \frac{N_{c}V_{c}}{{\left[\beta_{>,\mu }^{2}\gamma_{>,\mu }+\beta_{>,\nu }^{2}\gamma_{>,\nu }\right]}^{N}}\;{\left(\frac{\beta_{>,\mu }^{2}\gamma_{>,\mu }+\beta_{>,\nu }^{2}\gamma_{>,\nu }}{\beta_{<,\mu }^{2}\gamma_{<,\mu }+\beta_{<,\nu }^{2}\gamma_{<,\nu }}\right)}^{M}$$ (14)

Substituting the above into Eq (8) gives us,

$$\theta =\frac{\ln(2)}{\ln\left(2\right)+\left(c-1\right)\ln\;\left[\beta_{>,\mu }^{2}\gamma_{>,\mu }+\beta_{>,\nu }^{2}\gamma_{>,\nu }\right]-c\ln\;\left[\beta_{<,\mu }^{2}\gamma_{<,\mu }+\beta_{<,\nu }^{2}\gamma_{<,\nu }\right]}$$ (15)

where we have substituted *N*_*c*_ = 2^*N*^, and introduced the generation ratio, *c* = *M*/*N*, the ratio of the transitioning generation to the total number of generations. For a full derivation of Eq (15), see S4 Text.

**Fig 6:**
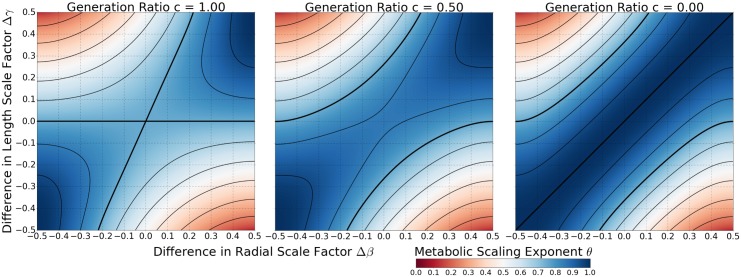
Colormap of Metabolic Scaling Exponent for a Network with a Transition in Flow Type. Here we present colormaps of Eq (15) for the cases when *c* = 1, *c* = 0.5, and *c* = 0. It should be noted that when the transition in flow type occurs within the networks, the same values for the *difference* scale factors are used, but the equations that determine the *average* scale factors switch from Eqs (16) and (17) to Eqs (20) and (21). In all three colormaps, the contour lines take on the same values as in Figs 4 and 5. In particular, the bolded contour corresponds to a metabolic scaling exponent value of 3/4.

Expressing Eq (15) in terms of the generation ratio allows for a convenient way to investigate the effects of a generation based transition in fluid flow type, and thus in the scale factors, but without having to specify the network size. When *c* = 1, the network has no transition and consists only of pulsatile flow, corresponding to the infinite mass limit discussed in [15]. On the other hand, when *c* = 0, the network again has no transition but now consists only of constant laminar flow, corresponding to the small mass limit discussed in both [9] and [15]. These limiting cases are presented in Fig 6, along with an intermediate case for when *c* = 0.5, or when the transition occurs halfway through the network. It is important to point out that in Fig 6 the networks are such that, both before and after the transition in fluid flow type, the same *difference* scale factors describe the network. What does change are the equations used to determine the corresponding values of the *average* scale factors. Thus, the colormaps corresponding *c* = 1 and *c* = 0 in Fig 6 are identical to Figs 4 and 5, respectively. To visualize the complete transition from *c* = 1 to *c* = 0 as an animation, see S2 Video.

Of particular importance in Fig 6, as well as in the animation provided in S2 Video, is the observation that 3/4 metabolic scaling can still be maintained, but at the direct expense of symmetric branching (note the change in location of the 3/4 contour). This seems to indicate that the inclusion of fluid transitions and finite-size-networks need not preclude precise 3/4 metabolic scaling, as discussed in previous work [15]. Even though we still make predictions for a metabolic scaling exponent of 1 in a constant laminar flow network based on strict morphological constraints, Eqs (18)–(21), the ability to branch asymmetrically in the pulsatile flow regime appears to provide for a means for maintaining 3/4 metabolic scaling in a network that exhibits a transition in fluid flow type.

### Nodal level results

Ensuring that the networks are characterized by space-filling fractal branching architectures that minimize dissipative effects associated with resource transport results in nodal level constraints between the scale factors. These constraints are both specific to, and shared between, the two fluid flow regimes. A summary of these results are presented in Fig 3.

#### Nodal level results for pulsatile flow

In the pulsatile flow regime we find that impedance matching across generations requires that the cross-sectional area must be preserved across individual bifurcations, $r_{j}^{2}=r_{j+1,\mu }^{2}+r_{j+1,\nu }^{2}$. When expressed in terms of the *average* and *difference* scale factors this result takes the form of,

$$1={\left(\beta_{j}+\Delta \beta_{j}\right)}^{2}+{\left(\beta_{j}-\Delta \beta_{j}\right)}^{2}$$ (16)

**Fig 7:**
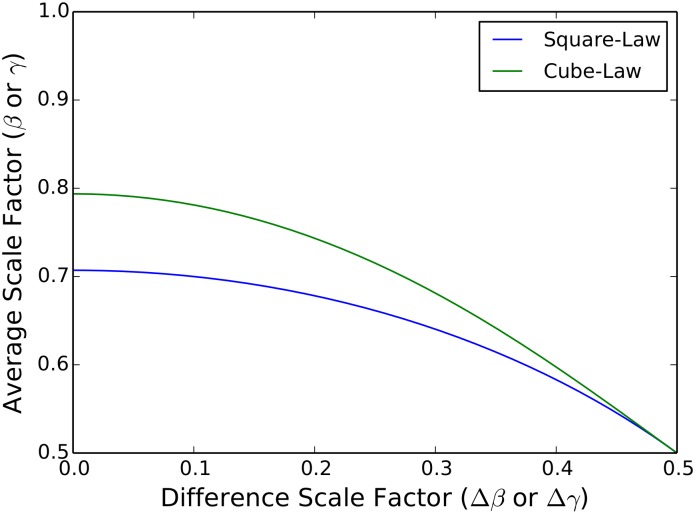
*Average* vs. *Difference* Scale Factors for Area-Preserving and Space-Filling/Area-Increasing Principles. The functional dependence between the *average* scale factors (*β*, *γ*) and the *difference* scale factors (Δ*β*, Δ*γ*) are graphed. For both cases of either the square-law (area-preserving) or the cube-law (space-filling and area-increasing) the *average* scale factors decrease from 1/2^1/2^ and 1/2^1/3^, respectively, and converge to 0.5 as the *difference* scale factors increase from 0 to 0.5.

Treating the blood as an incompressible fluid, the preservation of the cross-sectional area across bifurcations results in the blood velocity remaining constant through this portion of the network [40]. Furthermore, the preservation of the cross-sectional area throughout the pulsatile regime does not preclude covariation of the *average* and *difference* radial scale factors, *β*_*j*_ and Δ*β*_*j*_. That is, different values of the scale factors can occur across generations as long as Eq (16) is maintained. This is different from the symmertic WBE model result of *β* = 1/2^1/2^ ≈ 0.707 across all generations. It should be noted that in the limit that Δ*β*_*j*_ goes to 0, the asymmetric result converges to the symmetric result of *β* = 1/2^1/2^. Eq (16) is graphed in Fig 7, where the branching ratio *β*_*j*_ is shown to vary from 1/2^1/2^ ≈ 0.707 to 0.5 as the asymmetry ratio Δ*β*_*j*_ increases from 0 to 0.5.

The constraint that the networks be space-filling fractals with regard to the scaling of the lengths across generations results in $l_{j}^{3}=l_{j+1,\mu }^{3}+l_{j+1,\nu }^{3}$. When expressed in terms of the *average* and *difference* scale factors takes the form of,

$$1={\left(\gamma_{j}+\Delta \gamma_{j}\right)}^{3}+{\left(\gamma_{j}-\Delta \gamma_{j}\right)}^{3}$$ (17)

As with the radii, this relationship does not constrain the *average* and *difference* length scale factors, *γ*_*j*_ and Δ*γ*_*j*_ to take on singular values, but instead can covary with each other across generations. This result is also different from the symmetric WBE model result of *γ*_*j*_ = 1/2^1/3^ ≈ 0.793 across all generations. As with the radius, in the limit that Δ*γ*_*j*_ goes to 0, the asymmetric result converges to the symmetric result. This functional dependence between *γ*_*j*_ and Δ*γ*_*j*_ is also graphed in Fig 7, where *γ*_*j*_ is shown to vary from 1/2^1/3^ ≈ 0.793 to 0.5 as the asymmetry ratio Δ*γ*_*j*_ increases from 0 to 0.5.

Lastly, no restriction on asymmetry type is predicted by Eqs (16) and (17) for the pulsatile flow regime. That is, impedance matching and space-filling alone do not preclude the network from switching between asymmetry types from one generation to the next, or for that matter exhibiting a mix of asymmetry types within a given generation. The freedom of mixed asymmetry type has interesting implications for the self-similarity of the network. In particular, strict self-similarity would require no mixing of asymmetry types whatsoever. On the other hand, statistical self-similarity could still be exhibited in the presence of mixed asymmetry types [41, 42].

#### Nodal level results for constant laminar flow

In the constant laminar flow regime we find an assortment of strict constraints on the network morphology. These are resultant of the method of undetermined Lagrange multipliers, used to minimize the effects of resistance to fluid flow while ensuring that the network is a space-filling fractal. Specifically, we find: a selection for positive asymmetric branching over negative; a cubic-powered generational covariation between the *average* and *difference* radial scale factors, also known as Murray’s Law; the same cubic relationship between the *average* and *difference* length scale factors; equality of the *average* radial and length scale factors; and equality between the *difference* radial and length scale factors. These results can be presented by the following equations,

$$\beta_{j}=\gamma_{j}$$ (18)

$$\Delta \beta_{j}=\Delta \gamma_{j}$$ (19)

$$1={\left(\gamma_{j}+\Delta \gamma_{j}\right)}^{3}+{\left(\gamma_{j}-\Delta \gamma_{j}\right)}^{3}$$ (20)

$$1={\left(\beta_{j}+\Delta \beta_{j}\right)}^{3}+{\left(\beta_{j}-\Delta \beta_{j}\right)}^{3}$$ (21)

The selection of positive asymmetric branching over negative can be seen by fixing the value of Δ*β*_*j*_ to be greater then zero and considering the case where Δ*γ*_*j*_ ranges from positive to negative (the procedure necessary for transitioning from positive to negative asymmetry, as is diagrammed in Fig 1). In this scenario, when Δ*γ*_*j*_ < 0 we find a contradiction in the form of Δ*β*_*j*_ = −Δ*γ*_*j*_. Thus, negative asymmetric branching is suppressed and only positive asymmetric branching should occur in the constant laminar flow regime. This result can be better understood by considering the form of the resistance formula for Hagens-Poiseuille (constant laminar) flow. As $Z_{j}\propto l_{j}/r_{j}^{4}$, networks with a greater abundance of long, narrow vessels, as in the case of networks with repeated negative asymmetric branching, will experience a greater total resistance to flow.

In the symmetric WBE model Eq (18) is also found, only there the generational dependence is absent. In fact, in the symmetric limits, where Δ*β*_*j*_ and Δ*γ*_*j*_ tend to 0, Eqs (20) and (21) give rise to the symmetric WBE model results of *β* = *γ* = 1/2^1/3^ ≈ 0.783, as shown in Fig 7.

As discussed above, Eq (20) can be recognized as an asymmetric variation of Murray’s Law, but expressed in terms of scale factors [25]. Written in terms of the branch radii, Eq (20) takes the form,

$$r_{j}^{3}=r_{j+1,\mu }^{3}+r_{j+1,\nu }^{3}$$ (22)

With some rearrangement (see S2 Text), the above can be expressed in terms of the cross-sectional areas of the parent and child generations,

$$A_{j+1,\mu }+A_{j+1,\nu }=A_{j}\;\left(\frac{1}{\beta_{j}}-4\Delta \beta_{j}^{2}\right)$$ (23)

where $A_{j}=\pi r_{j}^{2}$. This way of expressing Murray’s Law is important as it allows us to examine how asymmetric branching influences fluid flow rates. In the symmetric limit, where Δ*β*_*j*_ = 0 and 1/*β*_*j*_ = 2^1/3^ ≈ 1.26, we find the symmetric WBE model result that cross-sectional area increases with each generation. Again, treating blood as an incompressible fluid, this results in a slowing of the flow rate across each generation. In the asymmetric limit, where Δ*β*_*j*_ = *β*_*j*_ = 0.5, the above equation reduces to Eq (16), and the cross-sectional area is constant across generations. This results in maintaining the speed of blood flow across generations. As can be seen in Fig 7, *β* strictly decreases as Δ*β* increases. Thus, we can infer that asymmetric branching provides for a mechanism for controlling the blood flow rate. An interesting consequence of this is that in the asymmetric limit for the radial scale factor, the metabolic scaling exponent for constant laminar flow takes on the 3/4 value (see Fig 5).

As with the pulsatile flow regime, the generational dependence of Eqs (20) and (21) imply that strict self-similarity is not predicted to be exhibited, although it may be exhibited statistically. Furthermore, not only are these results direct consequences from the Lagrange multiplier approach, but they are also consistent with the impedance matching conditions, but for the case of the Hagens-Poiseuille Law for constant laminar flow.

In Fig 7 we can see the relationship between the *average* and *difference* scale factors for constant laminar flow. As the values of the *difference* scale factors are increased from 0 to 0.5, the values of the *average* scale factors subsequently decrease from 1/2^1/3^ to 0.5 for both the radii scale factors and the length scale factors as they both follow the same cube-law.

To summarize, the method of undetermined Lagrange multipliers, used to determine the network parameters that minimize energy loss due to viscous friction in the constant laminar flow regime, predicts that negative asymmetric branchings violate energy minimization. Therefore, selection is expected to act against negative asymmetric branching under these scenarios and instead select for either positive asymmetric branching, or strict symmetric branching. Thus, the overall network architecture of the cardiovascular system is predicted to exhibit either type of asymmetric branching (positive or negative) in the pulsatile regime and only positive asymmetric branching in the constant laminar flow regime, with the potential for symmetric branching throughout. Within these flow regimes, the branches are further predicted to adhere to cross-sectional area preservation in the pulsatile regime and Murray’s cubic scaling law in the constant flow regime. Although the networks are still space-filling fractals, self-similarity is no longer predicted to be strictly adhered to in the presence of asymmetric branching. Here, “strict self-similarity” is interpreted as having constant values for the length scale factors both across and within generations. However, self-similarity may still exist at a statistical level where an average value for the length scale factors can be identified.

## Discussion

The theory we present makes specific predictions for how energy-minimizing and space-filling principles constrain the variation in network morphology and metabolic scaling exponents. The theory also shows that metabolic scaling exponents can deviate from the observed 3/4 value due to asymmetric branching. Conversely, we also show that there exists a wide array of asymmetric branching phenotypes that differ in their degree of asymmetric branching but, nonetheless, still show 3/4 metabolic scaling.

We show that branching asymmetry necessitates two flavors of candidate networks: positive asymmetry, where one child branch is both wider and longer than the other child branch; and negative asymmetry, where one child branch is wider but shorter than the other child branch. Noting that there are these two classes of networks, positive and negative, provides a straightforward method to classify the diversity of biological resource distribution networks. However, this classification is particular to only bifurcating branching networks. Previous work on asymmetric branching [35] suggests that our theory can be extended to trifurcations and even higher order branching furcation numbers.

Minimizing the cost of resource transport due to power dissipation leads to several predictions. Within the pulsatile flow regime it was found that area-preserving and space-filling branching at the nodal level are predicted, Eqs (16) and (17), and with no restrictions on either asymmetry type. This means that variation in the values of *β*_*j*_, Δ*β*_*j*_, *γ*_*j*_, and Δ*γ*_*j*_ can occur across generations, as well as a mixing of asymmetry types both within and across generations. Assuming no mixing of the asymmetry type, and that the scale factors are constant, leads to the aforementioned observations of the metabolic scaling exponent. Here, negative asymmetry leads to scaling values strictly less than 3/4, and positive asymmetry leads to scaling between approximately 0.5 and 1. Future work in considering the tradeoffs between achieving greater metabolic efficiency (scaling values associated with negative asymmetry) and bio-mechanical limitations may help to inform how mixed asymmetry and non-constant scale factors influence the metabolic scaling exponent.

Within the constant laminar flow regime, Murray’s Law and space-filling branching at the nodal level are predicted, Eqs (16) and (17), as well as the equality of the *average* scale factors for length and radius and similarly of the *difference* scale factors, Eqs (18) and (19). Additionally, it is predicted that only positive asymmetry is permitted. These results combine to show that the constant laminar flow regime will exhibit only a metabolic scaling exponent of 1, as is similarly predicted by the *symmetric* WBE model.

We combine these two fluid types into one network by presenting a third model in which the fluid flow transition from pulsatile to constant laminar occurs within one single generation. By allowing the transitioning generation to vary, we can smoothly shift from a network of complete pulsatile flow to one with complete constant laminar flow, where the intermediate regime consists of some mixture of both fluid flow types. Here we show, again, that 3/4 metabolic scaling can be achieved, but only in the presence of asymmetric branching. Thus, we rectify a standing problem in the *symmetric* WBE theory in which fluid flow transitions were shown to necessitate an increase in the predicted metabolic scaling exponent from 3/4.

The inability to make stricter predictions on the specific values of the scale factors is a consequence of having introduced additional variables to the model (Δ*β*_*j*_ and Δ*γ*_*j*_). This is a natural consequence of introducing additional variables without having introducing additional constraints. Nonetheless, we do provide for a mathematically rigorous framework with which to better describe and characterize already established patterns and hierarchies in vascular branching, in particular the human heart, mouse lung, and dorsal zone of the Zebrafish [26, 28, 29]. The search for additional constraints on additional variables, based on both physical and biological principles, is a natural future direction that could reveal important insights and be necessary to truly understand the architecture and flows of branching networks.

### Caveats

Our work highlights some important caveats and areas for future work. First, we have modeled asymmetric networks as consisting of strict self-similarity in branching rules. Specifically, the scale factors *β*_*j*_, *γ*_*j*_, Δ*β*_*j*_, and Δ*γ*_*j*_ are constant throughout the networks. This also assumes that the network is characterized by only one set of branching rules. It would be instructive to explore how relaxing the self-similarity assumption as well as allowing for the mixing of branching rules (asymmetry types) both across and within generations influences our results.

Second, finite-size effects in an asymmetric network are also of particular interest to explore. Some of the current (symmetric) allometric relationships rely on assumptions regarding the countability of the number of terminal network tips. Two examples of such assumptions are that all terminal tips are identical, and the relationship $N_{TIPS}=2^{N_{GEN}}$ for a strictly bifurcating, where *N*_*TIPS*_ represents the total number of terminal tips (capillaries) and *N*_*GEN*_ represents the total number of branching generations within the network. In an asymmetric network, these assumptions do not in general hold, as high degrees of asymmetry can lead to some branches reaching the terminal tip size sooner than others.

Third, asymmetric branching may provide a novel avenue for studying the flow transition regime within the cardiovascular system. Previous studies have modeled the transition as either a discrete step or a continuous shift, but in both cases symmetric branching was assumed [9, 15]. Asymmetric branching provides for transitions such that within a single generation there exists branches with both pulsatile and constant laminar flow. This level of mixing in flow types may provide for a more dynamical analysis of the flow transition regime.

### Conclusion

The WBE model provides a basis to understand the linkages between organism form and function. It has served as a platform for broad application to various other fields [6, 13, 16–18]. However, in its original form, the WBE model is built around a questionable assumption of symmetrical branching networks. As a consequence, it does not capture the great extent of variation in biological form. In biology, symmetric networks are rarely observed. Thus, a core assumption of the theory is violated. In this paper, we investigated if including asymmetry into the WBE model yields similar conclusions and predictions as the original model based on symmetric branching. Specifically, do optimal asymmetric networks follow 3/4 metabolic scaling?

We have derived a more general form of the WBE model. It incorporates different branching geometries reflected in differences in branching asymmetry. We believe that our approach can offer a more general theory that can better relate variation in organismal form and function than the original WBE model. Our definition of asymmetry in a strictly bifurcating network allows for a more accurate analysis of biological branching networks. In addition, the theory makes a set of novel predictions for the type of branching asymmetry favored under different fluid flow types/transfer regimes.

## Methods

### Determining the total network volume

We can first write the total volume of the *i*^*th*^ generation in terms of the total volume of the *k*^*th*^ generation, where *i* > *k*, as follows,

$$V_{i,TOT}=V_{k,TOT}\prod_{j=k}^{i-1}\;\left[\beta_{j,\mu }^{2}\gamma_{j,\mu }+\beta_{j,\nu }^{2}\gamma_{j,\nu }\right]$$ (24)

where the volume of a given branch is expressed as, $V_{j,\mu }=\pi r_{j,\mu }^{2}l_{j,\nu }$. The above expression becomes more transparent when setting *i* = 1 and *k* = 0. Using the definitions in Eq (4), the above reduces to an expression of how the volume in the base is split into two volume fractions for the two child branches. For larger values of *i* and *k*, the above expression can then be expanded to express *V*_i,TOT_ in terms of volume fractions of *V*_k,TOT_, where the fractions consist of different *k* to *i* path-dependent permutations. For example, when *i* = 2 and *k* = 0, there are four volume fractions for the four respective paths from the base branch to the four tips. It should be noted that Eq (24) already assumes that the scale factors within a given generation are the same. This does not however imply that all of the big child radii or lengths are equal within a given generation, and similarly for the small child radii and lengths.

Solving for, and summing over, the dummy generation-index *k*, and taking the limit that *i* goes to the *N*^*th*^ generation, will give us the total volume for an asymmetric network,

$$V_{TOT}=V_{N,TOT}\sum_{k=0}^{N}\;\left\{\prod_{j=k}^{N-1}\;{\left[\beta_{j,\mu }^{2}\gamma_{j,\mu }+\beta_{j,\nu }^{2}\gamma_{j,\nu }\right]}^{-1}\right\}$$ (25)

where *V*_*N*,*TOT*_ represents the combined volume of all of the terminal capillaries, and can be expressed as *V*_*N*,*TOT*_ = *N*_*C*_ *V*_*C*_. Imposing the assumption that the scale factors are the same across generations simplifies Eq (25) to,

$$V_{TOT}=\frac{N_{C}V_{C}}{{\left[\beta_{\mu }^{2}\gamma_{\mu }+\beta_{\nu }^{2}\gamma_{\nu }\right]}^{N}}\;\left\{\frac{1-{\left[\beta_{\mu }^{2}\gamma_{\mu }+\beta_{\nu }^{2}\gamma_{\nu }\right]}^{N+1}}{1-\left[\beta_{\mu }^{2}\gamma_{\mu }+\beta_{\nu }^{2}\gamma_{\nu }\right]}\right\}$$ (26)

A consequence of our first assumption, that the networks are space-filling fractals that minimize energy-loss, is that $\beta_{\mu }^{2}\gamma_{\mu }+\beta_{\nu }^{2}\gamma_{\nu }<1$, which allows us to approximate everything in the brackets in the above expression to a value of 1, to leading order. This can be interpreted as stating that the total volume in the network is decreasing from one generation to the next, and is consistent with Eqs (16, 17, 20) and (21). Upon making this approximation we arrive at,

$$V_{TOT}\approx \frac{N_{C}V_{C}}{{\left[\beta_{\mu }^{2}\gamma_{\mu }+\beta_{\nu }^{2}\gamma_{\nu }\right]}^{N}}$$ (27)

### Minimizing energy loss in space-filling fractal vascular networks

Having identified two candidates for asymmetric networks, we can impose energy minimization principles while simultaneously requiring that the networks be space-filling fractals for the two different flow regimes of constant laminar flow and pulsatile flow. Doing so allows for constraints on the extent to which asymmetry is exhibited. The underlying concept in these approaches is to determine which values of *β*_*j*_, *γ*_*j*_, Δ*β*_*j*_, and Δ*β*_*j*_ have been naturally selected for, through the process of evolution, such that the network transports resources with the least amount of resistance. As there are two different flow regimes, constant laminar and pulsatile, we use two different methods of energy minimization. In employing our different techniques for the different types of flow we are considering the flow regimes as independent from one another in that we do not incorporate transitioning between them. Although in naturally occurring organisms (i.e. ones with beating hearts) pulsatile flow dominates at lower-order generations where the vessel diameters are large, constant flow dominates at higher-order generations where the vessel diameters are small.

#### Pulsatile flow network

In the pulsatile flow regime of the network, we directly impose the space-filling constraint,

$$l_{j}^{3}=l_{j+1,\mu }^{3}+l_{j+1,\nu }^{3}$$ (28)

Eq (28) represents the physical constraint that the branch lengths scale as space-filling fractals. The exponent in Eq (28) is the Hausdorff dimension, *D*_H_, and is associated with the scaling of lengths across generations. In order for the lengths of the network to scale as space-filling fractals, this exponent must be equal to the Euclidean dimension, *D*_E_, of the space being filled. Thus, as a parent branch subdivides into two child branches, the exponent by which the lengths scale should equal 3. This condition has the interpretation of each branch in a given generation in the network being responsible for servicing the volumes of the distal branches, where the volumes are characterized either as spheres with diameter *l*_*k*_, or cubes with length *l*_*k*_ [9, 15, 43, 44].

We also impose impedance matching at the nodal level to minimize the power dissipated from interference effects due to the reflection of pressure waves at a branching junction. The impedance for pulsatile flow in a rigid cylinder is given by $Z_{j}=c_{0}^{2}\rho /\pi r_{j}^{2}c$, where *c*_0_ is the Kortweg-Moens velocity, *ρ* is the fluid density, and *c* is the wave velocity. As the child branches can be considered as being in parallel, then impedance matching across generations takes the form of,

$$\frac{1}{Z_{j}}=\frac{1}{Z_{j+1,\mu }}+\frac{1}{Z_{j+1,\nu }}$$ (29)

Note that secondary effects, such as the direction of the pressure-waves, are neglected in the above expression [45]. Impedance matching is biologically essential as having mis-matched impedances results in pulse reflections traveling toward, and interfering with, the heart.

#### Constant laminar flow

In examining the constant laminar flow regime of an organism, we use the method of undetermined Lagrange multipliers to minimize the power dissipated through the entire network by friction forces working against fluid flow. This technique allows for performing such a minimization while simultaneously maintaining fixed values for the total network volume and mass, as well as requiring that the branch lengths scale as a space-filling fractal.

In this regime we are assuming that the entire network experiences constant laminar flow. The fluid is assumed to follow the Hagens-Poiseuille Law for impedance to fluid flow in a rigid cylinder, given by $Z_{j}=8\mu l_{j}/\pi r_{j}^{4}$, where *μ* is the fluid viscosity [40]. The power dissipated throughout the entire network is given by $P={\dot{Q}}_{0}^{2}Z_{TOT}$, where ${\dot{Q}}_{0}$ is the volumetric flow rate through the aorta, and *Z*_TOT_ is the equivalent impedance of the entire network. In order to write down *Z*_TOT_, we start first with expressing the total impedance of the *i*^th^ generation in terms of the total impedance of the *k*^th^ generation, where *i* > *k*,

$$Z_{i,TOT}=Z_{k,TOT}\prod_{j=k}^{i-1}\;{\left[\frac{\beta_{j,\mu }^{4}}{\gamma_{j,\mu }}+\frac{\beta_{j,\nu }^{4}}{\gamma_{j,\nu }}\right]}^{-1}$$ (30)

Note that, in the above equation, the limit that *i* = 1 and *k* = 0 reduces the expression to that of the equivalent impedance of a parallel branching with two different impedances. For larger values of *i* and *k*, the above expression can be written as a sum of 2^*i*−*k*^ permutations of paths from the *k*^th^ generation to the *i*^th^ generation. To find the total impedance, *Z*_TOT_, we solve for *Z*_k_, and sum over all *k* generations from *k* = 0 to *k* = *i* in the limit that *i* goes to the *N*^th^ generation, arriving at,

$$Z_{TOT}=Z_{N,TOT}\sum_{k=0}^{N}\;\left\{\prod_{j=k}^{N-1}\;\left[\frac{\beta_{j,\mu }^{4}}{\gamma_{j,\mu }}+\frac{\beta_{j,\nu }^{4}}{\gamma_{j,\nu }}\right]\right\}$$ (31)

In order to minimize the power lost to friction against the constraints of a fixed total volume, a fixed total mass, and the requirement that the network is a space-filling fractal across each generation, we must minimize the objective function $F(\beta_{j,\mu },\beta_{j\nu },\gamma_{j,\mu },\gamma_{j,\nu })$ to variations in *β*_*j*,*μ*_, *β*_*j*,*ν*_, *γ*_*j*,*μ*_, and, *γ*_*j*,*ν*_. The objective function is defined as,

$$F={\dot{Q}}_{0}^{2}Z_{TOT}+\lambda V_{TOT}+\lambda_{M}M+\sum_{k=0}^{N}\;\left\{\lambda_{k}l_{N,TOT}^{3}\;\prod_{j=k}^{N-1}\;{\left[\gamma_{j,\mu }^{3}+\gamma_{j,\nu }^{3}\right]}^{-1}\right\}$$ (32)

where *Z*_TOT_ is defined in Eq (31), *V*_TOT_ is defined in Eq (25), and *M* is the total mass of the network. Here, *λ* and *λ*_*M*_ are the Lagrange multipliers that serve to fix the values of the total volume and mass, respectively. The coefficients *λ*_*k*_ are the Lagrange multipliers that serve to enforce the space-filling fractal condition across every generation, which is represented by the fourth term in Eq (32). The factor $l_{N,TOT}^{3}$ is equal to the sum of all of the cubed-lengths of the terminal tips in the *N*^th^ generation. The derivation of this fourth term can be done in the same manner as that taken for the total network volume and impedance.

The minimization procedure requires taking derivatives of F with respect to the various free parameters (*β*_*j*_, *γ*_*j*_, Δ*β*_*j*_, Δ*γ*_*j*_) and setting the resulting equations equal to zero to solve for the subsequent conditions on, or values of, *β*_*j*_, *γ*_*j*_, Δ*β*_*j*_, and Δ*γ*_*j*_. These calculations are highlighted in the supplementary information section S1 Text.

## Supporting information

S1 Text Lagrange Multipliers On Asymmetric Networks. (PDF)

S2 Text Asymmetric Branching and Murray’s Law. (PDF)

S3 Text Symmetric/Difference Formalism of Asymmetric Branching. (PDF)

S4 Text Deriving the Metabolic Scaling Exponent for an Asymmetric Vascular Network with a Sharp Generational Transition. (PDF)

S1 Fig Colormap of Metabolic Scaling Exponent for Pulsatile Flow Using Symmetric/Difference Formalism. (PDF)

S1 Video Evolution of Asymmetric Network Morphology Along 3/4 Metabolic Scaling Exponent Contour for Pulsatile Flow. (MPG)

S2 Video Animated Colormap of Metabolic Scaling Exponent for an Asymmetric Vascular Network with a Sharp Generational Transition. (MP4)
