# Supplementary material for: A general model for metabolic scaling in self-similar asymmetric networks
Source: PLoS Comput Biol. 2017 Mar 20;13(3):e1005394. doi: 10.1371/journal.pcbi.1005394 (PMC5378416; doi:10.1371/journal.pcbi.1005394)
Supplement: S1 Text — (PDF) [file pcbi.1005394.s001.pdf]

S1 Text

Lagrange Multipliers On Asymmetric Networks

Minimizing $\mathcal{F}$ With Respect To Radius Ratios.

The objective function that we are to minimize is,

$$\mathcal{F} = \dot{Q}_0^2 Z_{N,TOT} \sum_{k=0}^{N} \left\{ \prod_{j=k}^{N-1} \left[\frac{\beta_{j,\mu}^4}{\gamma_{j,\mu}} + \frac{\beta_{j,\nu}^4}{\gamma_{j,\nu}} \right] \right\} + \lambda V_{N,TOT} \sum_{k=0}^{N} \left\{ \prod_{j=k}^{N-1} \left[\beta_{j,\mu}^2 \gamma_{j,\mu} + \beta_{j,\nu}^2 \gamma_{j,\nu} \right]^{-1} \right\}$$
$$+ \lambda_M M + \sum_{k=0}^{N} \left\{ \lambda_k l_{N,TOT}^3 \prod_{j=k}^{N-1} \left[\gamma_{j,\mu}^3 + \gamma_{j,\nu}^3 \right]^{-1} \right\} \quad (1)$$

Varying $\mathcal{F}$ with respect to $\beta_{i,\mu}$, by evaluating $\partial\mathcal{F}/\partial\beta_{i,\mu} = 0$, gives,

$$0 = \sum_{k=0}^{i} \left\{ \frac{4\dot{Q}_0^2 Z_{N,TOT} \beta_{i,\mu}^3}{\gamma_{1,\mu}} \prod_{\substack{j=k \\ j\neq i}}^{N-1} \left[\frac{\beta_{j,\mu}^4}{\gamma_{j,\mu}} + \frac{\beta_{j,\nu}^4}{\gamma_{j,\nu}} \right] - \frac{2\lambda V_{N,TOT} \beta_{i,\mu}\gamma_{i,\mu}}{(\beta_{i,\mu}^2\gamma_{i,\mu} + \beta_{i,\nu}^2\gamma_{i,\nu})^2} \prod_{\substack{j=k \\ j\neq i}}^{N-1} \left[\beta_{j,\mu}^2 \gamma_{j,\mu} + \beta_{j,\nu}^2 \gamma_{j,\nu} \right]^{-1} \right\} \quad (2)$$

Varying $\mathcal{F}$ with respect to $\beta_{i,\nu}$, by evaluating $\partial\mathcal{F}/\partial\beta_{i,\nu} = 0$, gives,

$$0 = \sum_{k=0}^{i} \left\{ \frac{4\dot{Q}_0^2 Z_{N,TOT} \beta_{i,\nu}^3}{\gamma_{1,\nu}} \prod_{\substack{j=k \\ j\neq i}}^{N-1} \left[\frac{\beta_{j,\mu}^4}{\gamma_{j,\mu}} + \frac{\beta_{j,\nu}^4}{\gamma_{j,\nu}} \right] - \frac{2\lambda V_{N,TOT} \beta_{i,\nu}\gamma_{i,\nu}}{(\beta_{i,\mu}^2\gamma_{i,\mu} + \beta_{i,\nu}^2\gamma_{i,\nu})^2} \prod_{\substack{j=k \\ j\neq i}}^{N-1} \left[\beta_{j,\mu}^2 \gamma_{j,\mu} + \beta_{j,\nu}^2 \gamma_{j,\nu} \right]^{-1} \right\} \quad (3)$$

The above two equations can be combined to arrive at,

$$0 = \sum_{k=0}^{i} \left\{ 4\dot{Q}_0^2 Z_{N,TOT} \prod_{j=k}^{N-1} \left[\frac{\beta_{j,\mu}^4}{\gamma_{j,\mu}} + \frac{\beta_{j,\nu}^4}{\gamma_{j,\nu}} \right] - 2\lambda V_{N,TOT} \prod_{j=k}^{N-1} \left[\beta_{j,\mu}^2 \gamma_{j,\mu} + \beta_{j,\nu}^2 \gamma_{j,\nu} \right]^{-1} \right\} \quad (4)$$

As the above equality holds for any i and for all k, then we can set each term within the summation to zero,

$$0 = 4\dot{Q}_0^2 Z_{N,TOT} \prod_{j=k}^{N-1} \left[\frac{\beta_{j,\mu}^4}{\gamma_{j,\mu}} + \frac{\beta_{j,\nu}^4}{\gamma_{j,\nu}} \right] - 2\lambda V_{N,TOT} \prod_{j=k}^{N-1} \left[\beta_{j,\mu}^2 \gamma_{j,\mu} + \beta_{j,\nu}^2 \gamma_{j,\nu} \right]^{-1} \quad (5)$$

From here we can solve for λ to find,

$$\lambda = \frac{2\dot{Q}_0^2 R_N}{V_{N,TOT}} \prod_{j=k}^{N-1} \left[\left(\frac{\beta_{j,\mu}^4}{\gamma_{j,\mu}} + \frac{\beta_{j,\nu}^4}{\gamma_{j,\nu}} \right) \left(\beta_{j,\mu}^2 \gamma_{j,\mu} + \beta_{i,\mu}^2 \gamma_{i,\mu} \right) \right] \quad (6)$$

Substituting the above expression for λ into either $\partial\mathcal{F}/\partial\beta_{i,\mu} = 0$, or $\partial\mathcal{F}/\partial\beta_{i,\nu} = 0$, gives $\gamma_{j,\nu}/\gamma_{j,\mu} = \beta_{j,\nu}/\beta_{j,\mu}$. Additionally, being that λ is a constant quantity, our result must hold for all values of k. Setting $\lambda|_k = \lambda|_{k+1}$ leads to,

$$1 = \beta_{j,\mu}^6 + \beta_{j,\mu}^4 \beta_{j,\nu}^2 \frac{\gamma_{j,\nu}}{\gamma_{j,\mu}} + \beta_{j,\nu}^4 \beta_{j,\mu}^2 \frac{\gamma_{j,\mu}}{\gamma_{j,\nu}} + \beta_{j,\nu}^6 \tag{7}$$

Upon substitution of $\gamma_{j,\nu}/\gamma_{j,\mu} = \beta_{j,\nu}/\beta_{j,\mu}$, the above expression reduces to,

$$1 = \beta_{j,\mu}^3 + \beta_{j,\nu}^3 \tag{8}$$

which is the familiar Murray's Law of cubic-powered radial scaling, but in the context of asymmetric branching. Note that, unlike the symmetric result of $\beta = 1/2^{1/3}$, different values of β and $\Delta\beta$ at each branching point may be exhibited as long as Murray's Law is still maintained. In other words, self-similarity in the radial dimension is not strictly maintained from one generation to the next, although it may be still be exhibited stochastically.

Minimizing $\mathcal{F}$ With Respect To Length Ratios.

Varying $\mathcal{F}$ with respect to $\gamma_{i,\mu}$ and $\gamma_{i,\nu}$, by evaluating $\partial\mathcal{F}/\partial\gamma_{i,\mu} = 0$ and $\partial\mathcal{F}/\partial\gamma_{i,\nu} = 0$, leads to,

$$0 = \sum_{k=0}^{i} \Bigg\{ -\dot{Q}_0^2 Z_{N,TOT} \prod_{j=k}^{N-1} \left[\frac{\beta_{j,\mu}^4}{\gamma_{j,\mu}} + \frac{\beta_{j,\nu}^4}{\gamma_{j,\nu}} \right] - \lambda V_{N,TOT} \prod_{j=k}^{N-1} \left[\beta_{j,\mu}^2 \gamma_{j,\mu} + \beta_{j,\nu}^2 \gamma_{\text{j},\nu} \right]^{-1} \\ -3\lambda_k l_{N,TOT}^3 \prod_{j=k}^{N-1} \left[\gamma_{j,\mu}^3 + \gamma_{j,\nu}^3 \right]^{-1} \Bigg\} \tag{9}$$

Substituting our earlier expression for λ results in,

$$0 = \sum_{k=0}^{i} \left\{ -3\dot{Q}_0^2 Z_{N,TOT} \prod_{j=k}^{N-1} \left[\frac{\beta_{j,\mu}^4}{\gamma_{j,\mu}} + \frac{\beta_{j,\nu}^4}{\gamma_{j,\nu}} \right] - 3\lambda_k l_{N,TOT}^3 \prod_{j=k}^{N-1} \left[\gamma_{j,\mu}^3 + \gamma_{j,\nu}^3 \right]^{-1} \right\} \tag{10}$$

Again, as the above equation holds for any value i and for all k, then we can set each term within the summation to zero and solve for λ_k to arrive at,

$$\lambda_k = -\frac{\dot{Q}_0^2 Z_{N,TOT}}{l_{N,TOT}^3} \prod_{j=k}^{N-1} \left\{ \left[\frac{\beta_{j,\mu}^4}{\gamma_{j,\mu}} + \frac{\beta_{j,\nu}^4}{\gamma_{j,\nu}} \right] \left[\gamma_{j,\mu}^3 + \gamma_{j,\nu}^3 \right] \right\} \tag{11}$$

As done before for λ, we can substitute the above expression for λ_k into either $\partial\mathcal{F}/\partial\gamma_{i,\mu} = 0$ or $\partial\mathcal{F}/\partial\gamma_{i,\nu} = 0$ also results in $\gamma_{j,\nu}/\gamma_{j,\mu} = \beta_{j,\nu}/\beta_{j,\mu}$, the same relationship found before from minimizing $\mathcal{F}$ with respect to the radii. Furthermore, as $\lambda_k = k + 1$, we have,

$$1 = \left[\frac{\beta_{j,\mu}^4}{\gamma_{j,\mu}} + \frac{\beta_{j,\nu}^4}{\gamma_{j,\nu}} \right] \left[\gamma_{j,\mu}^3 + \gamma_{j,\nu}^3 \right] \tag{12}$$

Using the conditions $\beta_{j,\mu}/\beta_{j,\nu} = \gamma_{j,\mu}/\gamma_{j,\nu}$ and $1 = \beta_{j,\mu}^3 + \beta_{j,\nu}^3$, the above expression results in $\gamma_{j,\nu} = \beta_{j,\nu}$ and $\gamma_{j,\mu} = \beta_{j,\mu}$. These equalities between the length and radius scale factors have a significant interpretation regarding the whole network architecture.

First, when substituted into our asymmetric variation of Murray's Law, Eq. (8), we find that,

$$1 = \gamma_{j,\mu}^3 + \gamma_{j,\nu}^3 \tag{13}$$

which is the asymmetric version of space-filling exhibited at the nodal level. Eq. (13) differs from the symmetric result of $\gamma = 1/2^{1/3}$, which means that, like our asymmetric variation of Murray's Law, self-similarity in the length dimension is not strictly adhered to. Although, self-similarity may be stochastically exhibited. It should also be noted that, for Hagens-Pouiseille flow, the cubic-law relationships for the length and radii scale factors, Eqs. (8) and (13), also satisfy impedance matching.

Secondly, when combining the definitions of the scale factors with the results of $\gamma_{j,\nu} = \beta_{j,\nu}$ and $\gamma_{j,\mu} = \beta_{j,\mu}$ we find that $\gamma_j = \beta_j$ and $\Delta\gamma_j = \Delta\beta_j$. Recalling that the switch from the positive asymmetry network to negative asymmetry network involves fixing $\Delta\gamma$ to fall within the domain of $[0, 0.5)$ while restricting $\Delta\beta$ to the domain of $(-0.5, 0]$, we find a contradiction with the result that $\Delta\gamma_j = \Delta\beta_j$, except for the symmetric limit in which all of these conditions are satisfied. In other words, the method of undetermined Lagrange multipliers, used to determine the network parameters that minimizes energy loss due to viscous friction in the constant laminar flow regime, predicts that negative asymmetry branchings violate energy minimization, and therefore ought to be suppressed in favor of either positive asymmetric, or strict symmetric, branching. Thus, the overall network architecture of the cardiovascular system is predicted to exhibit either type of asymmetric branching (positive or negative) in the pulsatile regime and only positive asymmetry branching in the constant laminar flow regime, with the potential for symmetric branching throughout. Within these flow regimes, the branches are further predicted to simultaneously adhere to nodal variations of cross-sectional area preservation and space-filling (pulsatile), and nodal variations of Murray's Law and space-filling (constant laminar).

Showing the proportionality of total volume to mass.

Substituting our expression for λ_k back into the objective function $\mathcal{F}$ results in,

$$\mathcal{F} = \lambda V_{TOT} + \lambda_M M \tag{14}$$

where we have written the total network volume as V_{TOT}. To examine how total network volume, V_{TOT}, varies with mass M, we vary the objective function $\mathcal{F}$ with respect to mass by evaluating $\partial\mathcal{F}/\partial M = 0$ to arrive at,

$$0 = \lambda \frac{\partial V_{TOT}}{\partial M} + \lambda_M \tag{15}$$

Solving for $\partial V_{TOT}/\partial M$ gives,

$$\frac{\partial V_{TOT}}{\partial M} = -\frac{\lambda_M}{\lambda} \tag{16}$$

Given that the lagrange multipliers are constants, we can finally solve for V_{TOT} with using the separation of variables to show that $V_{TOT} \propto M$ for an asymmetrically bifurcating vascular network.
