# Supplementary material for: A general model for metabolic scaling in self-similar asymmetric networks
Source: PLoS Comput Biol. 2017 Mar 20;13(3):e1005394. doi: 10.1371/journal.pcbi.1005394 (PMC5378416; doi:10.1371/journal.pcbi.1005394)
Supplement: S2 Text — (PDF) [file pcbi.1005394.s002.pdf]

S2 Text

Asymmetric Branching and Murray's Law

In symmetric branching, Murray's Law of cubic scaling for branch radii has the implication of a strict increase in the total cross-sectional area of the child branches when compared to the parent branch. For constant laminar flow of an incompressible fluid, this strict increase in area results in the slowing down of fluid flow, a necessary condition in the cardiovascular system a blood flow rates in the capillaries are slower than those in the arteries. Our interest stems in how the introduction of branching asymmetry influences the increase in cross-sectional area, and thus the subsequent slowing of blood flow.

Recalling the form of Murray's Law as expressed in Eq. (20) of the main text, we have,

$$r_j^3 = r_{j+1,\mu}^3 + r_{j+1,\nu}^3 \tag{1}$$

To examine how asymmetric branching influences the generational scaling of cross-sectional area, we can re-write Eq. (1) in terms of the cross-sectional areas associated with each branch,

$$A_j r_j = A_{j+1,\mu} r_{j+1,\mu} + A_{j+1,\nu} r_{j+1,\nu} \tag{2}$$

where $A_j = \pi r_j^2$ is the cross-sectional area of a branch with radius r_j. Substituting the definitions for the scale factors from Eq. (4) of the main text, and solving for A_j gives

$$A_j = A_{j+1,\mu}\beta_{j,\mu} + A_{j+1,\nu}\beta_{j,\nu} \tag{3}$$

Expressing $\beta_{j,\mu}$ and $\beta_{j,\nu}$ in terms of the *average* and *difference* scale factors, we can write the cross-sectional area of the parent branch, A_j, in terms of the sum and difference of the cross-sectional areas of the child branches,

$$A_j = (A_{j+1,\mu} + A_{j+1,\nu})\beta_j + (A_{j+1,\mu} - A_{j+1,\nu})\Delta\beta_j \tag{4}$$

From here, we can now solve for the total cross-sectional area of the child generation to examine its dependence on asymmetric branching. Doing so, we have,

$$A_{j+1,\mu} + A_{j+1,\nu} = \frac{1}{\beta_j}\left\{A_j + \left[A_{j+1,\nu} - A_{j+1,\mu}\right]\Delta\beta_j\right\} \tag{5}$$

The cross-sectional areas of the child branches on the right-hand-side of the above equation can be expressed in terms of the *averages* and *differences* in the child radii to arrive at,

$$A_{j+1,\mu} + A_{j+1,\nu} = \frac{1}{\beta_j}\left\{A_j - 4\pi r_{j+1}\Delta r_{j+1}\Delta\beta_j\right\} \tag{6}$$

Recalling the definitions for the *average* and *difference* radial scale factors, $\beta_j = r_{j+1}/r_j$ and $\Delta\beta_j = \Delta r_{j+1}/r_j$ respectively, allows us to simplify the above expression to,

$$A_{j+1,\mu} + A_{j+1,\nu} = \frac{1}{\beta_j}A_j - 4\pi\Delta r_{j+1}^2 \tag{7}$$

As we are interested in examining how asymmetric branching influences the area-increasing interpretation of Murray's Law, we can factor $A_j = \pi r_j^2$ from the right hand side of the above expression to arrive at,

$$A_{j+1,\mu} + A_{j+1,\nu} = A_j \left(\frac{1}{\beta_j} - 4\Delta\beta_j^2 \right) \tag{8}$$

In the symmetric limit, where $\Delta\beta_j = 0$ and $1/\beta_j = 2^{1/3}$, Eq. (8) reduces to the symmetric WBE model result where the cross-sectional area increases across each generation, from parent to child, by a factor of approximately 1.26. However, in the asymmetric limit, where $\Delta\beta_j = 0.5$ and $\beta_j = 0.5$, Eq. (8) reduces to $A_{j+1,\mu} + A_{j+1,\nu} = A_j$. That is, cross-sectional area is preserved across generations. As β_j monotonically decreases as a function of $\Delta\beta_j$ (see Fig. 6 in the main text), we can conclude that there is a steady transition from increasing cross-sectional area to constant cross-sectional area as radial asymmetry is increased in the constant laminar flow regime.

The dependance of Eq. (8) on $\Delta\beta_j$ is significant because it demonstrates the ability of asymmetric branching to control the flow rate of blood. Treating blood as an incompressible fluid, then Eq. (8) indicates that maximal symmetry results in the the greatest rate of increase in cross-sectional area across a bifurcation, which in turn causes the blood flow to slow down at the greatest rate. On the other hand, maximal asymmetry results in constant cross-sectional area across a bifurcation, which in turn maintains blood flow at a constant speed across branching.
