# Supplementary material for: A general model for metabolic scaling in self-similar asymmetric networks
Source: PLoS Comput Biol. 2017 Mar 20;13(3):e1005394. doi: 10.1371/journal.pcbi.1005394 (PMC5378416; doi:10.1371/journal.pcbi.1005394)
Supplement: S3 Text — (PDF) [file pcbi.1005394.s003.pdf]

S3 Text

Symmetric/Difference Formalism of Asymmetric Branching

An alternative to the *average/difference* formalism for characterizing asymmetry is to perturb off of the *symmetric* WBE formalism. In this approach, the physical scale factors are expressed in terms of the *symmetric* WBE scale factors and perturbations from those values. Starting with an analogous form of Eq. (2) in the main text, we can write the physical lengths and radii of a pair of sibling branches as,

$$\begin{aligned} l_{j,\mu} &= \tilde{l}_{\mathrm{WBE},\ j} + \Delta\tilde{l}_{j,\mu} & \qquad l_{j,\nu} &= \tilde{l}_{\mathrm{WBE},\ j} - \Delta\tilde{l}_{j,\nu} \\ r_{j,\mu} &= \tilde{r}_{\mathrm{WBE},\ j} + \Delta\tilde{r}_{j,\mu} & \qquad r_{j,\nu} &= \tilde{r}_{\mathrm{WBE},\ j} - \Delta\tilde{r}_{j,\nu} \end{aligned} \tag{1}$$

We can express the above in terms of scale factors to arrive at,

$$\begin{aligned} \gamma_{j,\mu} &= \tilde{\gamma}_{\mathrm{WBE}} + \Delta\tilde{\gamma}_{j,\mu} & \qquad \gamma_{j,\nu} &= \tilde{\gamma}_{\mathrm{WBE}} - \Delta\tilde{\gamma}_{j,\nu} \\ \beta_{j,\mu} &= \tilde{\beta}_{\mathrm{WBE}} + \Delta\tilde{\beta}_{j,\mu} & \qquad \beta_{j,\nu} &= \tilde{\beta}_{\mathrm{WBE}} - \Delta\tilde{\beta}_{j,\nu} \end{aligned} \tag{2}$$

where $\tilde{\beta}_{\mathrm{WBE}}$ and $\tilde{\gamma}_{\mathrm{WBE}}$ are the *symmetric* WBE scale factors and $\Delta\tilde{\gamma}_{j,\mu}$, $\Delta\tilde{\gamma}_{j,\nu}$, $\Delta\tilde{\beta}_{j,\mu}$ and $\Delta\tilde{\beta}_{j,\nu}$ are the *symmetric-difference* scale factors. It should be noted that the *symmetric* scale factors have fixed values, where $\tilde{\beta}_{\mathrm{WBE}} = (1/2)^{1/2}$ for pulsatile flow and $(1/2)^{1/3}$ for constant laminar flow, and $\tilde{\gamma}_{\mathrm{WBE}} = (1/2)^{1/3}$ for both types of flow. However, all four of the *symmetric-difference* scale factors are free to vary. This approach is beneficial in that it allows for results to be expressed strictly in terms of deviations from the *symmetric* WBE results. However, it does not as easily distinguish between positive and negative type asymmetry, and in certain circumstances may even obscure the presence (or absence) of asymmetry all together. For example, should both child branches have physical length scale factors of 0.8, then the *symmetric-difference* length scale factors would have non-zero values of $\Delta\tilde{\gamma}_{j,\mu} = 0.8 - \tilde{\gamma}_{\mathrm{WBE}}$ and $\Delta\tilde{\gamma}_{j,\nu} = \tilde{\gamma}_{\mathrm{WBE}} - 0.8$.

When imposing the constraints that result from space-filling and minimizing energy loss, specific domains can be placed on the *symmetric-difference* scale factors. These domains are $\Delta\tilde{\beta}_{\mu} \in [-\tilde{\beta}_{\mathrm{WBE}}, 1 - \tilde{\beta}_{\mathrm{WBE}}]$, $\Delta\tilde{\beta}_{\nu} \in [\tilde{\beta}_{\mathrm{WBE}} - 1, \tilde{\beta}_{\mathrm{WBE}}]$, $\Delta\tilde{\gamma}_{\mu} \in [-\tilde{\gamma}_{\mathrm{WBE}}, 1 - \tilde{\gamma}_{\mathrm{WBE}}]$, $\Delta\tilde{\gamma}_{\nu}[\tilde{\gamma}_{\mathrm{WBE}} - 1, \tilde{\gamma}_{\mathrm{WBE}}]$, and can be derived by comparing the two definitions of the physical scale factors (*average/difference* and *symmetric/difference*) in the limits of complete symmetry or asymmetry. It should be noted that these limits on the *symmetric-difference* scale factors produce the same limits for the physical scale factors $(\beta_{\mu}, \beta_{\nu}, \gamma_{\mu}, \gamma_{\nu}) \in [0, 1]$.

Under the same assumptions made in the text regarding asymmetry within the total network, the metabolic scaling exponent can be expressed four different ways depending on which choice of substitutions for the physical scale factors is made. For example, if we wanted to examine the metabolic scaling exponent as a function of $\Delta\beta_{\mu}$ and $\Delta\gamma_{\mu}$, we would begin by substituting into the general expression for the metabolic scaling exponent (Eq. (13) in the main text) $\beta_{\nu}^2 = 1 - \beta_{\mu}^2$ and $\gamma_{\nu} = (1 - \gamma_{\mu}^3)^{1/3}$ to explicitly incorporate the constraints due to energy minimization and space-filling (and remove all β_{ν} and γ_{ν} dependence). Next, substituting the perturbative definitions for β_{μ} and γ_{μ} from Eq. (2), and with some rearranging, we can arrive at the following expression for the metabolic scaling exponent,

$$\theta_{\mu\mu} = -\Bigg\{ \frac{\ln(\tilde{\beta}_{\rm WBE}^2 \tilde{\gamma}_{\rm WBE})}{\ln(2)} + \frac{1}{\ln(2)} \ln \Bigg[\frac{1}{2\tilde{\beta}_{\rm WBE}^2} \left(\frac{1}{\tilde{\gamma}_{\rm WBE}^3} - \left(1 + \frac{\Delta\tilde{\gamma}_\mu}{\tilde{\gamma}_{\rm WBE}}\right)^3 \right)^{1/3}$$

$$+ \frac{1}{2} \left(1 + \frac{\Delta\tilde{\beta}_\mu}{\tilde{\beta}_{\rm WBE}}\right)^2 \left(\left(1 + \frac{\Delta\tilde{\gamma}_\mu}{\tilde{\gamma}_{\rm WBE}}\right) - \left(\frac{1}{\tilde{\gamma}_{\rm WBE}^3} - \left(1 + \frac{\Delta\tilde{\gamma}_\mu}{\tilde{\gamma}_{\rm WBE}}\right)^3 \right)^{1/3} \right) \Bigg] \Bigg\}^{-1} \quad (3)$$

Performing all possible permutations of substitutions provides us with three more expressions for the metabolic scaling exponent, each in terms of two of the four possible *symmetric-difference* scale factors.

$$\theta_{\nu\nu} = -\Bigg\{ \frac{\ln(\tilde{\beta}_{\rm WBE}^2 \tilde{\gamma}_{\rm WBE})}{\ln(2)} + \frac{1}{\ln(2)} \ln \Bigg[\frac{1}{2\tilde{\beta}_{\rm WBE}^2} \left(\frac{1}{\tilde{\gamma}_{\rm WBE}^3} - \left(1 - \frac{\Delta\tilde{\gamma}_\nu}{\tilde{\gamma}_{\rm WBE}}\right)^3 \right)^{1/3}$$

$$+ \frac{1}{2} \left(1 - \frac{\Delta\tilde{\beta}_\nu}{\tilde{\beta}_{\rm WBE}}\right)^2 \left(\left(1 - \frac{\Delta\tilde{\gamma}_\nu}{\tilde{\gamma}_{\rm WBE}}\right) - \left(\frac{1}{\tilde{\gamma}_{\rm WBE}^3} - \left(1 - \frac{\Delta\tilde{\gamma}_\nu}{\tilde{\gamma}_{\rm WBE}}\right)^3 \right)^{1/3} \right) \Bigg] \Bigg\}^{-1} \quad (4)$$

$$\theta_{\mu\nu} = -\Bigg\{ \frac{\ln(\tilde{\beta}_{\rm WBE}^2 \tilde{\gamma}_{\rm WBE})}{\ln(2)} + \frac{1}{\ln(2)} \ln \Bigg[\frac{1}{2\tilde{\beta}_{\rm WBE}^2} \left(1 - \frac{\Delta\tilde{\gamma}_\nu}{\tilde{\gamma}_{\rm WBE}}\right)$$

$$+ \frac{1}{2} \left(1 + \frac{\Delta\tilde{\beta}_\mu}{\tilde{\beta}_{\rm WBE}}\right)^2 \left(\left(\frac{1}{\tilde{\gamma}_{\rm WBE}^3} - \left(1 - \frac{\Delta\tilde{\gamma}_\nu}{\tilde{\gamma}_{\rm WBE}}\right)^3 \right)^{1/3} - \left(1 - \frac{\Delta\tilde{\gamma}_\nu}{\tilde{\gamma}_{\rm WBE}}\right) \right) \Bigg] \Bigg\}^{-1} \quad (5)$$

$$\theta_{\nu\mu} = -\Bigg\{ \frac{\ln(\tilde{\beta}_{\rm WBE}^2 \tilde{\gamma}_{\rm WBE})}{\ln(2)} + \frac{1}{\ln(2)} \ln \Bigg[\frac{1}{2\tilde{\beta}_{\rm WBE}^2} \left(1 + \frac{\Delta\tilde{\gamma}_\mu}{\tilde{\gamma}_{\rm WBE}}\right)$$

$$+ \frac{1}{2} \left(1 - \frac{\Delta\tilde{\beta}_\nu}{\tilde{\beta}_{\rm WBE}}\right)^2 \left(\left(\frac{1}{\tilde{\gamma}_{\rm WBE}^3} - \left(1 + \frac{\Delta\tilde{\gamma}_\mu}{\tilde{\gamma}_{\rm WBE}}\right)^3 \right)^{1/3} - \left(1 + \frac{\Delta\tilde{\gamma}_\mu}{\tilde{\gamma}_{\rm WBE}}\right) \right) \Bigg] \Bigg\}^{-1} \quad (6)$$

In all four of the above equations we can see that variation in the metabolic scaling exponent depends primarily on variations in length. The first term is exactly that which produces the 3/4 value of the *symmetric* WBE model. The first term in the square brackets represents deviations from 3/4 metabolic scaling due strictly to variations in the scaling of length (as denoted by the presence of $\Delta\tilde{\gamma}_\mu$ or $\Delta\tilde{\gamma}_\nu$), while the second term in the square brackets depends on both variations in the scaling of length and radius (as denoted by the presence of both of the *symmetric-difference* scale factors for length and radius. Also present in all four equations is the tendency of the metabolic scaling exponent to take on a value of 3/4 for all cases where there is zero asymmetry in length. For each of the expressions, the first term in the square brackets will reduce to a value of 1, while the second term in the square brackets will reduce to a value of 0, all upon substitution of $\tilde{\beta}_{WBE} = 1/2^{1/2}$ and $\tilde{\gamma}_{WBE} = 1/2^{1/3}$. In this limit we can see that the metabolic scaling exponent θ will not change from 3/4 even when variation in the scaling of the radii is present.

Each of the expressions has an associated color map presented, respectively, in S1 Fig. **A**-**D**. Of note is that the general behavior of the color maps is similar to one other, as well as to that produced with the *average/difference* formalism, in terms of regions of increase and decrease in the value of the metabolic scaling exponent from 3/4 (the red and blue sections of the color maps). This similarity has been highlighted by including the same contours of constant metabolic scaling

exponent in each color map, as well as by demarcating the locations in the color maps that correspond to the asymmetric trees in Fig. 5 of the main text.

The appearance of the color maps to be non-linear transformations of the color map from the *average/difference* formalism can be understood by examining the functional form of the coordinate transformations from the *average/difference* formalism to the *symmetric/difference* formalism. Upon inspection of the definitions for the physical scale factors in terms of the two different formalisms, we can solve for the *symmetric-difference* scale factors in terms of the *average* and *difference* scale factors to arrive at,

$$\begin{aligned} \Delta\tilde{\gamma}_\mu &= \gamma + \Delta\gamma + \tilde{\gamma}_{\text{WBE}} \qquad & \Delta\tilde{\gamma}_\nu &= \tilde{\gamma}_{\text{WBE}} + \Delta\gamma - \gamma \\ \Delta\tilde{\beta}_\mu &= \beta + \Delta\beta + \tilde{\beta}_{\text{WBE}} \qquad & \Delta\tilde{\beta}_\nu &= \tilde{\beta}_{\text{WBE}} + \Delta\beta - \beta \end{aligned} \tag{7}$$

While the above transformations may appear linear, we must recall that the space-filling and energy-minimizing constraints enforce non-linear relationships between β and $\Delta\beta$ as well as between γ and $\Delta\gamma$. Focusing on the radial scale factors, we can use the relationship $1 = (\beta_j + \Delta\beta_j)^2 + (\beta_j - \Delta\beta_j)^2$ to express β in terms of $\Delta\beta$ as $\beta = \sqrt{1/2 - \Delta\beta^2}$. Substituting this into the radial scale factor transformations in Eq. (7) results in

$$\Delta\tilde{\beta}_\mu = \sqrt{1/2 - \Delta\beta^2} + \Delta\beta + \tilde{\beta}_{\text{WBE}} \qquad \Delta\tilde{\beta}_\nu = \tilde{\beta}_{\text{WBE}} + \Delta\beta - \sqrt{1/2 - \Delta\beta^2} \tag{8}$$

Thus, we can clearly see the non-linearity between the *difference* scale factor $\Delta\beta$ and the *symmetric-difference* scale factors $\Delta\beta_\mu$ and $\Delta\beta_\nu$. A similar non-linear transformation for the length scale factors can be shown, but is omitted due to the burdensome nature of its form.
