# Supplementary material for: A general model for metabolic scaling in self-similar asymmetric networks
Source: PLoS Comput Biol. 2017 Mar 20;13(3):e1005394. doi: 10.1371/journal.pcbi.1005394 (PMC5378416; doi:10.1371/journal.pcbi.1005394)
Supplement: S4 Text — (PDF) [file pcbi.1005394.s004.pdf]

S4 Text

Deriving the Metabolic Scaling Exponent for an Asymmetric Vascular Network with a Sharp Generational Transition

Here we present the derivation for the metabolic scaling exponent in a vascular network that exhibits asymmetric branching and a transition in the values of the scale factors that occurs in a given branching generation. Within generations the scale factors do not change, however. For generality, we will start with assuming that inter-generational variation in the scale factors exists, while intra-generational variation does not. The latter assumption will not change throughout this document. We begin with Eq. (9) from the main text. Assuming that $i > k$, we can express the total volume of the ith generation in terms of the kth generation as,

$$V_{i,TOT} = V_{k,TOT} \prod_{j=k}^{i-1} \left[\beta_{j,\mu}^2 \gamma_{j,\mu} + \beta_{j,\nu}^2 \gamma_{j,\nu}\right] \tag{1}$$

Solving for $V_{\mathrm{k,TOT}}$, summing from $k = 0$ to $k = N$, where N is the maximum number of generations in the network, and setting $i = N$, we can write an expression for the total volume in the network as,

$$V_{TOT} = V_{N,TOT} \sum_{k=0}^{N} \left\{ \prod_{j=k}^{N-1} \left[\beta_{j,\mu}^2 \gamma_{j,\mu} + \beta_{j,\nu}^2 \gamma_{j,\nu}\right]^{-1} \right\} \tag{2}$$

To model a sharp generational transition, we will first re-write the above expression as two summations; the first running from $k = 0$ to $k = M$, and the second from $k = M + 1$ to $k = N$.

$$V_{TOT} = V_{M,TOT} \sum_{k=0}^{M} \left\{ \prod_{j=k}^{M-1} \left[\beta_{j,\mu}^2 \gamma_{j,\mu} + \beta_{j,\nu}^2 \gamma_{j,\nu}\right]^{-1} \right\} + V_{N,TOT} \sum_{k=M+1}^{N} \left\{ \prod_{j=k}^{N-1} \left[\beta_{j,\mu}^2 \gamma_{j,\mu} + \beta_{j,\nu}^2 \gamma_{j,\nu}\right]^{-1} \right\} \tag{3}$$

Imposing the assumption that the scale factors are constant across generations, we can remove the product over j and replace it with the appropriate exponent. However, to keep track of the two sets of scale factors that exists before and after generation M, we will adopt the naming convention used in West et al. Science 1997, where the less than symbol, $<$, used as a subscript, represents scale factors preceding the transition, and the greater than symbol, $>$, used as a subscript, represents scale factors after the transition. Under these assumptions and notations, the total volume of network takes the form of,

$$V_{TOT} = V_{M,TOT} \sum_{k=0}^{M} \left[\beta_{<,\mu}^2 \gamma_{<,\mu} + \beta_{<,\nu}^2 \gamma_{<,\nu}\right]^{-(M-k)} + V_{N,TOT} \sum_{k=M+1}^{N} \left[\beta_{>,\mu}^2 \gamma_{>,\mu} + \beta_{>,\nu}^2 \gamma_{>,\nu}\right]^{-(N-k)} \tag{4}$$

By setting $m = M - k$ and $n = N - k$, we can reset the dummy indices to simplify the above expression to the following,

$$V_{TOT} = V_{M,TOT} \sum_{m=0}^{M} \left[\beta_{<,\mu}^2 \gamma_{<,\mu} + \beta_{<,\nu}^2 \gamma_{<,\nu}\right]^{-m} + V_{N,TOT} \sum_{n=0}^{N-M-1} \left[\beta_{>,\mu}^2 \gamma_{>,\mu} + \beta_{>,\nu}^2 \gamma_{>,\nu}\right]^{-n} \tag{5}$$

Recognizing the above expressions as geometric series, we can remove the summations using the formula $\sum_{k=a}^{b} r^k = \frac{r^a - r^{b+1}}{1-r}$, assuming that $r \neq 1$. The condition of $r \neq 1$ can be physically

interpreted as meaning that volume is strictly not preserved across generations, but in fact varies. Making the appropriate replacements results in the following,

$$V_{TOT} = V_{M,TOT}\left\{\frac{1-\left[\beta^2_{<,\mu}\gamma_{<,\mu}+\beta^2_{<,\nu}\gamma_{<,\nu}\right]^{-(M+1)}}{1-\left[\beta^2_{<,\mu}\gamma_{<,\mu}+\beta^2_{<,\nu}\gamma_{<,\nu}\right]^{-1}}\right\}+V_{N,TOT}\left\{\frac{1-\left[\beta^2_{>,\mu}\gamma_{>,\mu}+\beta^2_{>,\nu}\gamma_{>,\nu}\right]^{-(N-M)}}{1-\left[\beta^2_{>,\mu}\gamma_{>,\mu}+\beta^2_{>,\nu}\gamma_{>,\nu}\right]^{-1}}\right\} \tag{6}$$

With some rearrangement, the above can be expressed in a slightly more intuitive manner that allows for easier approximation,

$$\begin{aligned} V_{TOT} = &\frac{V_{M,TOT}}{\left[\beta^2_{<,\mu}\gamma_{<,\mu}+\beta^2_{<,\nu}\gamma_{<,\nu}\right]^{M}}\left\{\frac{1-\left[\beta^2_{<,\mu}\gamma_{<,\mu}+\beta^2_{<,\nu}\gamma_{<,\nu}\right]^{(M+1)}}{1-\left[\beta^2_{<,\mu}\gamma_{<,\mu}+\beta^2_{<,\nu}\gamma_{<,\nu}\right]}\right\} \\ &+\frac{V_{N,TOT}}{\left[\beta^2_{>,\mu}\gamma_{>,\mu}+\beta^2_{>,\nu}\gamma_{>,\nu}\right]^{N}}\left\{\left(\frac{1-\left[\beta^2_{>,\mu}\gamma_{>,\mu}+\beta^2_{>,\nu}\gamma_{>,\nu}\right]^{(N+1)}}{1-\left[\beta^2_{>,\mu}\gamma_{>,\mu}+\beta^2_{>,\nu}\gamma_{>,\nu}\right]}\right)-\left(\frac{1-\left[\beta^2_{>,\mu}\gamma_{>,\mu}+\beta^2_{>,\nu}\gamma_{>,\nu}\right]^{(M+1)}}{1-\left[\beta^2_{>,\mu}\gamma_{>,\mu}+\beta^2_{>,\nu}\gamma_{>,\nu}\right]}\right)\right\} \end{aligned} \tag{7}$$

Now we need to express $V_{M,TOT}$ in terms of $V_{N,TOT}$ by again using Eq. (9) from the main text, but now for the case where $N > M$, taking the form of,

$$V_{N,TOT} = V_{M,TOT}\prod_{j=M}^{N-1}\left[\beta^2_{j,\mu}\gamma_{j,\mu}+\beta^2_{j,\nu}\gamma_{j,\nu}\right] \tag{8}$$

Since the set of scale factors $\{\beta_{j,\mu},\gamma_{j,\mu},\beta_{j,\nu},\gamma_{j,\nu}\}$ are fixed to be equal to $\{\beta_{>,\mu},\gamma_{>,\mu},\beta_{>,\nu},\gamma_{>,\nu}\}$ for $M \leq j \leq N$, then we can remove the product from the above expression and solve for $V_{\mathrm{M,TOT}}$ to arrive at,

$$V_{M,TOT} = V_{N,TOT}\left[\beta^2_{>,\mu}\gamma_{>,\mu}+\beta^2_{>,\nu}\gamma_{>,\nu}\right]^{M-N} \tag{9}$$

Substituting this expression into V_{TOT}, we have,

$$\begin{aligned} V_{TOT} = &\frac{V_{N,TOT}}{\left[\beta^2_{>,\mu}\gamma_{>,\mu}+\beta^2_{>,\nu}\gamma_{>,\nu}\right]^{N}}\left\{\left(\frac{\beta^2_{>,\mu}\gamma_{>,\mu}+\beta^2_{>,\nu}\gamma_{>,\nu}}{\beta^2_{<,\mu}\gamma_{<,\mu}+\beta^2_{<,\nu}\gamma_{<,\nu}}\right)^{M}\left(\frac{1-\left[\beta^2_{<,\mu}\gamma_{<,\mu}+\beta^2_{<,\nu}\gamma_{<,\nu}\right]^{(M+1)}}{1-\left[\beta^2_{<,\mu}\gamma_{<,\mu}+\beta^2_{<,\nu}\gamma_{<,\nu}\right]}\right)\right. \\ &\left.+\left(\frac{1-\left[\beta^2_{>,\mu}\gamma_{>,\mu}+\beta^2_{>,\nu}\gamma_{>,\nu}\right]^{(N+1)}}{1-\left[\beta^2_{>,\mu}\gamma_{>,\mu}+\beta^2_{>,\nu}\gamma_{>,\nu}\right]}\right)-\left(\frac{1-\left[\beta^2_{>,\mu}\gamma_{>,\mu}+\beta^2_{>,\nu}\gamma_{>,\nu}\right]^{(M+1)}}{1-\left[\beta^2_{>,\mu}\gamma_{>,\mu}+\beta^2_{>,\nu}\gamma_{>,\nu}\right]}\right)\right\} \end{aligned} \tag{10}$$

At this point we will make several assumptions to simplify the above expression. The first is that the network is large, or $N >> 1$. Additionally is that the transition in the scale factors occurs closer to the terminal end of the network, such that $M >> 1$. Lastly, we assume that the scale factor values are such that, for any given bifurcation, the volumes of the child branches sum to be strictly less than the volume of the corresponding parent branch. This last assumption translates into the two following inequalities, $\beta^2_{>,\mu}\gamma_{>,\mu}+\beta^2_{>,\nu}\gamma_{>,\nu} < 1$, and $\beta^2_{<,\mu}\gamma_{<,\mu}+\beta^2_{<,\nu}\gamma_{<,\nu} < 1$. These four assumptions allows us to approximate each the three quotients with the form of $\frac{1-x^b}{1-x}$, to be on the order unity, where $x = \beta^2_{>,\mu}\gamma_{>,\mu}+\beta^2_{>,\nu}\gamma_{>,\nu}$ or $x = \beta^2_{<,\mu}\gamma_{<,\mu}+\beta^2_{<,\nu}\gamma_{<,\nu}$, and $b = N+1$ or $b = M+1$. Alternatively phrased, the quantity within the curly brackets is dominated by the ratio $\left(\frac{\beta^2_{>,\mu}\gamma_{>,\mu}+\beta^2_{>,\nu}\gamma_{>,\nu}}{\beta^2_{<,\mu}\gamma_{<,\mu}+\beta^2_{<,\nu}\gamma_{<,\nu}}\right)^{M}$. Thus, we can simplify the above expression to take the form of,

$$V_{TOT} \approx \frac{V_{N,TOT}}{\left[\beta^2_{>,\mu}\gamma_{>,\mu} + \beta^2_{>,\nu}\gamma_{>,\nu}\right]^N} \left(\frac{\beta^2_{>,\mu}\gamma_{>,\mu} + \beta^2_{>,\nu}\gamma_{>,\nu}}{\beta^2_{<,\mu}\gamma_{<,\mu} + \beta^2_{<,\nu}\gamma_{<,\nu}}\right)^M \tag{11}$$

We can now use the proportionality relationship between total volume and total mass and substitute our expression for the total volume into the relationship for the metabolic scaling exponent as a function of body mass, $\theta = \frac{\ln(N_c)}{\ln(M/M_0)}$, and replacing $V_{N,TOT}$ with $N_c V_c$ (replacing the total volume of the Nth generation with the volume of a single capillary times the number of capillaries), gives us,

$$\theta = \frac{\ln(N_c)}{\ln\left\{\frac{N_c\left[\beta^2_{>,\mu}\gamma_{>,\mu}+\beta^2_{>,\nu}\gamma_{>,\nu}\right]^{M-N}}{\left[\beta^2_{<,\mu}\gamma_{<,\mu}+\beta^2_{<,\nu}\gamma_{<,\nu}\right]^M}\right\}} \tag{12}$$

Using *log*-arithmetic rules, we can express the above in a more intuitive form as,

$$\theta = \frac{\ln(N_c)}{\ln(N_c) + (M-N)\ln\left[\beta^2_{>,\mu}\gamma_{>,\mu} + \beta^2_{>,\nu}\gamma_{>,\nu}\right] - M\ln\left[\beta^2_{<,\mu}\gamma_{<,\mu} + \beta^2_{<,\nu}\gamma_{<,\nu}\right]} \tag{13}$$

In Eq. 13 we can parameterize the metabolic scaling of a network with a sharp transition by the number of terminal tips, N_{c}, the total number of generations in the network, N, the generation at which the transition occurs, M, and the sets of scale factors that describe the network architecture before and after the transition. To graph Eq 13 in such a way as to understand how varying the transition generation affects the metabolic scaling exponent landscape, Eq. 13 can be re-written by first substituting $N_c = 2^N$, and then factoring out the variable N, giving,

$$\theta = \frac{\ln(2)}{\ln(2) + (c-1)\ln\left[\beta^2_{>,\mu}\gamma_{>,\mu} + \beta^2_{>,\nu}\gamma_{>,\nu}\right] - c\ln\left[\beta^2_{<,\mu}\gamma_{<,\mu} + \beta^2_{<,\nu}\gamma_{<,\nu}\right]} \tag{14}$$

where $c = M/N$. Thus when $c = 1$ the entire network is described by the pre-transition scale factors, when $c = 0$ the entire network is described by the post-transition scale factors, and for any other values of c the network is described by a mixture. An array of colormaps have been made for values of c varying from $[0, 1]$, then turned into an animation for easier visualization (see **S2 Video**). It should be pointed out that in these colormaps a specific morphological form of the networks has been assumed. When the transition in flow type occurs within the networks, the same values for the *difference* scale factors are used, but the equations that determine the *average* scale factors switch from Eqs. (16) and (17) to Eqs. (18c) and (18d) with respect to the main text.
