# Supplementary material for: A general model for metabolic scaling in self-similar asymmetric networks
Source: PLoS Comput Biol. 2017 Mar 20;13(3):e1005394. doi: 10.1371/journal.pcbi.1005394 (PMC5378416; doi:10.1371/journal.pcbi.1005394)
Supplement: S1 Fig — (PDF) [file pcbi.1005394.s005.pdf]

S1 Fig

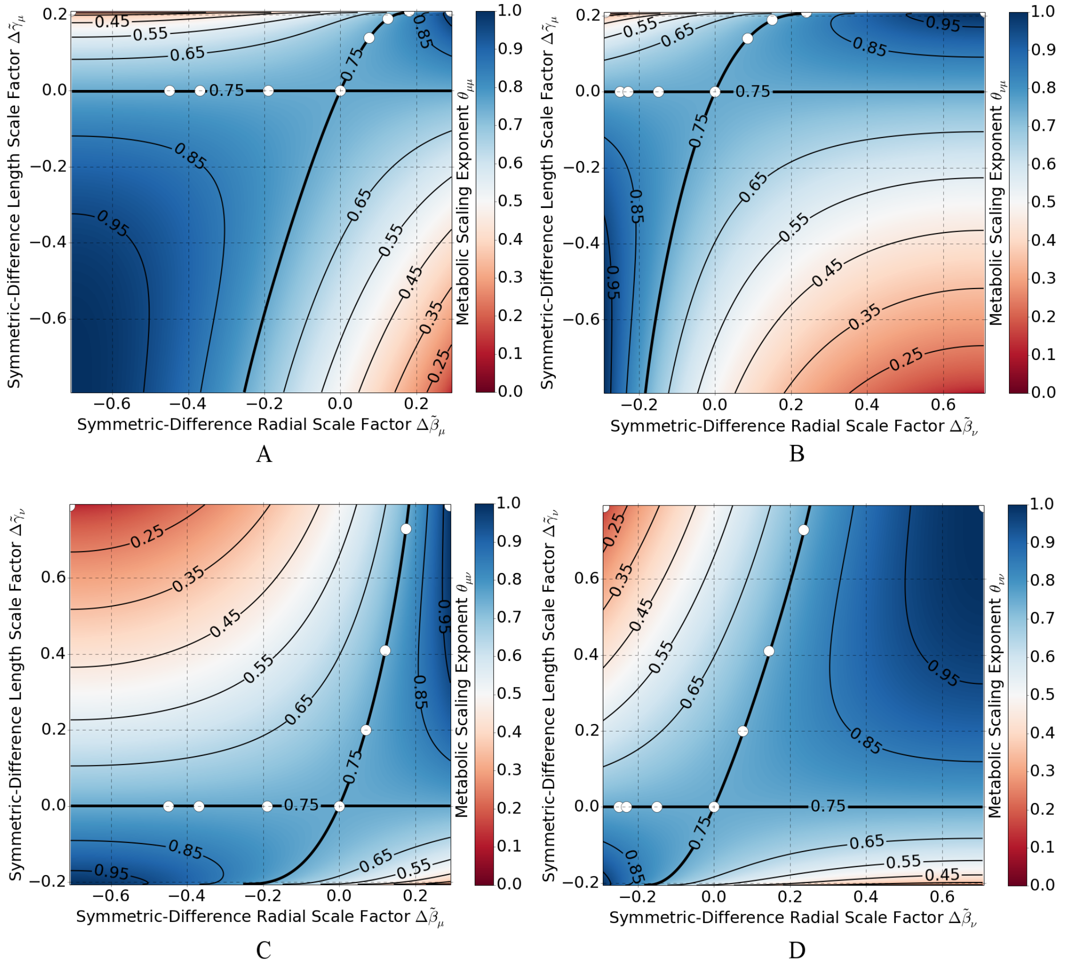

Figure 1. Colormap of Metabolic Scaling Exponent for Pulsatile Flow Using Symmetric/Difference Formalism

The metabolic scaling exponent is graphed as a function of the *symmetric-difference* scale factors $\Delta\beta_\mu, \Delta\beta_\nu, \Delta\gamma_\mu$, and $\Delta\gamma_\nu$ and is shown to range in value from 0 to 1. The scale factors are such that the networks are space-filling fractals that minimize energy-loss from resource transport. Contours of constant values of the metabolic scaling exponent are plotted in bold. The demarcated points correspond to the rendered trees found in Fig. 5 of the main text, and have the same ordering as those found in Fig. 4.
